# Dramatic Improvement of Proteomic Analysis of Zebrafish Liver Tumor by Effective Protein Extraction with Sodium Deoxycholate and Heat Denaturation

**DOI:** 10.1155/2015/763969

**Published:** 2015-03-19

**Authors:** Jigang Wang, Yew Mun Lee, Caixia Li, Ping Li, Zhen Li, Teck Kwang Lim, Zhiyuan Gong, Qingsong Lin

**Affiliations:** Department of Biological Sciences, National University of Singapore, 14 Science Drive 4, Singapore 117543

## Abstract

Majority of the proteomic studies on tissue samples involve the use of gel-based approach for profiling and digestion. The laborious gel-based approach is slowly being replaced by the advancing in-solution digestion approach. However, there are still several difficulties such as difficult-to-solubilize proteins, poor proteomic analysis in complex tissue samples, and the presence of sample impurities. Henceforth, there is a great demand to formulate a highly efficient protein extraction buffer with high protein extraction efficiency from tissue samples, high compatibility with in-solution digestion, reduced number of sample handling steps to reduce sample loss, low time consumption, low cost, and ease of usage. Here, we evaluated various existing protein extraction buffers with zebrafish liver tumor samples and found that sodium deoxycholate- (DOC-) based extraction buffer with heat denaturation was the most effective approach for highly efficient extraction of proteins from complex tissues such as the zebrafish liver tumor. A total of 4,790 proteins have been identified using shotgun proteomics approach with 2D LC, which to our knowledge is the most comprehensive study for zebrafish liver tumor proteome.

## 1. Introduction

It is increasingly important to profile proteins in order to understand biological processes in a postgenomic era as the dynamics of proteins between cells at different times and under different environmental conditions provide an actual biological phenotype. In particular, the presence of posttranslational modifications in proteins further highlights the importance of proteomic analysis which is not replaceable by other genomic approaches [1]. To profile the proteome of tissue samples, the proteins have to be extracted using relevant solvent. Currently, there are two major approaches to prepare the tissue samples for proteome analysis. The first approach, termed as gel-based separation and in-gel digestion, involves the use of detergents like SDS to solubilize the proteins before separation by SDS-PAGE and subsequent digestion of the proteins trapped in the gel [2]. The second approach, termed as in-solution digestion, involves the use of strong chaotropic reagents like urea and thiourea to solubilize the proteins before digesting the proteins in the solution [3].

Proteomic studies on zebrafish liver tissue had been conducted using the gel-based approaches [4–9]. However, the amount of work required from two-dimensional gel electrophoresis, to protein spot excision, to protein identification using mass spectrometry (MS) can be laborious. In-solution digestion coupled with mass spectrometry (MS) seems to present a better alternative to reduce the labor involved and allow for more high-throughput proteomic analysis. More studies are beginning to adopt this approach for proteomic analysis [10–12]. However, numerous difficulties still exist such as difficult-to-solubilize proteins, poor proteomic analysis in complex tissue samples, and the presence of sample impurities.

In 2009, Wiśniewski et al. [13] developed a protein extraction approach and coined it the filter-aided sample preparation (FASP). FASP incorporates the advantages of both gel-based and in-solution digestion for subsequent proteomic analysis using MS. FASP uses high concentration of SDS and urea as detergents to solubilize the proteins. This resulted in the need to use an ultrafiltration system consisting of a filter membrane and facilitated via centrifugation to remove these detergents as they are known to interfere with both enzymatic digestion of proteins into peptides and MS analysis. Although the authors demonstrated the effectiveness of FASP in proteomic analysis, multiple steps were included for the removal of the detrimental detergents. These could create unforeseen problems for different types of samples. Hence, there is still a great demand to formulate a highly efficient protein extraction buffer with high protein extraction efficiency from tissue samples, high compatibility with in-solution digestion, reduced number of sample handling steps to reduce sample loss, low time consumption, low cost, and ease of usage.

In this study, we evaluated various existing protein extraction buffers (SDS, RIPA, urea, 2D, sodium deoxycholate (DOC), and triethylammonium bicarbonate (TEAB)/urea/triton-X/SDS (TUTS) [14] buffer) for their protein extraction and solubilization efficiency for in-solution digestion using both 1D SDS-PAGE and shotgun proteomics approaches. Comparison of efficiency using these approaches indicated that DOC was the most efficient protein extraction buffer in our study. Our results provide the evidences for the effective application of DOC-based protein extraction buffer in MS-based proteomic studies on the whole zebrafish liver tumor and our method could be applied to other tissue samples across different organisms for proteomics analysis.

## 2. Materials and Methods

### 2.1. Chemicals and Reagents

All reagents were of ACS grade or higher; all solvents used, including water, were of LC/MS grade. Urea, SDS, DOC, triethylammonium bicarbonate (TEAB), tris(2-carboxyethyl)phosphine (TCEP), phosphoric acid, and formic acid were purchased from Sigma-Aldrich (St. Louis, MO). Sequencing grade trypsin was obtained from Promega (Madison, WI). Methyl methanethiosulfonate (MMTS) was purchased from Pierce, Thermo-Fisher Scientific Inc. (Rockford, IL). Unless otherwise indicated, all the other reagents used for the biochemical methods were purchased from Sigma-Aldrich. LC/MS grade ACN and LC/MS grade water were purchased from Thermo-Fisher Scientific (Waltham, MA).

### 2.2. Zebrafish Sample Preparation

The Tet-on transgenic zebrafish,* TO (xmrk)*, were generated previously by Li et al. [15]. Both male and female* TO (xmrk) *zebrafish were used in this study. The* TO (xmrk) *zebrafish were treated with 60 *μ*g/mL of doxycycline for 6 weeks to induce the development of liver cancer. The tumor-bearing fish were euthanized in ice-cold water, dissected, and rinsed with phosphate buffered saline (PBS). Liver tumors were collected and stored at −80°C until protein extraction. For protein extraction, frozen liver tumors were placed in a ceramic mortar and ground into dry powder using a pestle in the presence of acetone. Equal amount (10 mg) of tissue powder was transferred to seven Eppendorf tubes containing equal volume (200 *μ*L) of different protein extraction buffers (Table 1) and placed on ice. Each tube of the mixtures was sonicated using a probe sonicator at 2 s sonication bursts with a 2 s rest between each sonication burst for a total of 1 min. The lysate was then centrifuged in a bench-top centrifuge at 14,000x rpm for half an hour. Each of the supernatants was collected for 1D SDS-PAGE or for in-solution digestion. Samples were aliquoted and stored at −80°C for later analysis.

### 2.3. 1D SDS-PAGE Comparison of Zebrafish Liver Tumor Proteome Extracted Using Various Buffers

For SDS buffer and DOC buffer, both raw (without heating) and heat-denatured samples were prepared. The heat denaturation involves heating the samples on a 95°C heat block for 15 min after sonication. The lysates were then centrifuged in a bench-top centrifuge at 14,000x rpm for half an hour and collected for downstream analysis. Equal volumes (5 *μ*L) of protein samples extracted by different buffer were mixed with 5 *μ*L of 2x SDS loading buffer. The samples were heated to 95°C for 10 min and then separated by SDS gel electrophoresis with 10% polyacrylamide gel. After 1D SDS-PAGE, gels were stained using CBB solution (Bio-Rad, Hercules, CA) and destained in accordance with the manufacturer's protocol.

### 2.4. Liver Tumor Proteome In-Solution Digestion

Protein concentration of each lysate was determined using Bradford assay (Bio-Rad). Twenty-microgram samples (extracted by DOC buffer or SDS buffer) were used for the following in-solution digestion. Samples were diluted using 0.5 M TEAB to lower the detergent concentration in order to maintain the activity of the trypsin (0.05% SDS or 1% DOC does not affect the trypsin activity [16, 17]). pH was measured and adjusted to eight for trypsin digestion. The sample was subsequently reduced with 5 mM TCEP in a 65°C heat block for 60 min and alkylated with 10 mM MMTS for 15 min at room temperature. Following reduction and alkylation, trypsin (1 *μ*g, Promega; protein versus trypsin ratio: 20/1) was added and the sample incubated at 37°C overnight on a thermoshaker. The digested peptides were stored at −20°C pending LC separation and MS analysis.

### 2.5. Sample Cleanup before LC-MS/MS

Before the LC-tandem MS (LC-MS/MS) analysis, the sample must be cleaned up to remove the detergent, dissolution buffer (TEAB), reducing agent (TCEP), alkylating agent (MMTS), SDS, and other unknown interfering substances. To remove most of the DOC, 0.1% TFA was added into the DOC sample. After acidifying the sample, DOC was precipitated and removed by centrifugation at 14,000 rpm for 20 min. Then the DOC sample was subjected to strong cation-exchange chromatography (SCX) using the iTRAQ Method Development Kit (AB SCIEX; Foster City, CA). The bound peptides were eluted with 5% ammonium hydroxide (NH_4_OH) in 30% methanol. The eluate was desalted using a Sep-Pak C18 cartridge (Waters, Milford, MA), dried, and then reconstituted with 100 *μ*L of diluent (98% water, 2% ACN, and 0.05% formic acid). For the SDS lysed sample, it was also cleaned up by SCX and Sep-Pak C18 cartridge.

### 2.6. Protein Identification and Quantification

The detailed methods for LC-MS/MS were described previously [18]. Briefly, separation of the peptides was carried out on an Eksigent nanoLC Ultra and ChiPLC-nanoflex (Eksigent, Dublin, CA) in Trap-Elute configuration. Five-microliter samples were loaded onto the LC system. Peptides were separated by a gradient formed by 2% ACN, 0.1% FA (mobile phase A), and 98% ACN, 0.1% FA (mobile phase B): 5–12% of mobile phase B (20 min), 12–30% of mobile phase B (90 min), 30–90% of mobile phase B (2 min), 90% of mobile phase B (5 min), 90–5% of mobile phase B (3 min), and 5% of mobile phase B (13 min), at a flow rate of 300 nL/min. The MS analysis was performed on a TripleTOF 5600 system (AB SCIEX) in information dependent mode.

The detailed method of ProteinPilot analysis was described previously [18]. Briefly, the protein identification was performed with ProteinPilot 4.5 (AB SCIEX) which uses the Paragon algorithm to perform database searches. The database used includes the International Protein Index (IPI) v3.87 zebrafish protein sequences. The search parameters used were as follows: cysteine alkylation of MMTS; trypsin digestion; TripleTOF 5600; and biological modifications. Redundancy was eliminated by the grouping of identified proteins using the ProGroup algorithm in the software. A decoy database search strategy was used to determine the false discovery rate for peptide identification. A corresponding randomized database was generated using the Proteomics System Performance Evaluation Pipeline feature in the ProteinPilot Software 4.5. In this study, a strict unused score cut-off ≥1.3 was adopted as the qualification criterion, which corresponded to a peptide confidence level of ≥95%. The identification results were then exported into Microsoft Excel for manual data analysis.

### 2.7. Gene Ontology and Pathway Analysis

The identified proteins were subjected to gene ontology (GO) analysis using Software Tool for Rapid Annotation of Proteins (STRAP) v1.5.0.0 [19]. The pathway analysis was performed using Ingenuity Pathway Analysis (IPA) software v21249400 (Qiagen, Hilden, Germany). The figures generated were abstracted from IPA.

## 3. Results and Discussion

### 3.1. 1D SDS-PAGE Showed Better Protein Extraction Efficiency with SDS and DOC

In order to maximize the efficiency in the protein extraction and solubilisation of the liver tumors for downstream analysis, we compared various extraction buffers commonly used in laboratories worldwide (Table 1). Of these, SDS was chosen because it is one of the most common surfactants used to assist in the solubilisation of proteins, especially membrane proteins, during protein extraction [12, 13, 20]. Urea, a chaotrope, is another commonly used protein solubilizing agent that competes with the protein's native interactions, resulting in the unfolding of the protein and its solubilization [21]. In addition, urea is also found in TUTS buffer (one of the buffers tested in our study) which was formulated for a previous study on the subcellular localization of membrane proteins [14]. Furthermore, thiourea, a component in the 2D extraction buffer, is found to be a stronger denaturant than urea [22]. Henceforth, the 2D extraction buffer was also included in our study. DOC is an inexpensive bile salt surfactant which has been used in studies of membrane proteins [23, 24] and was included in our study due to its comparative protein extraction efficiency as SDS [23]. Lastly, RIPA is another commonly used protein extraction buffer in proteomic studies, and it contains a small percentage of both SDS and DOC.

**Figure 1:**
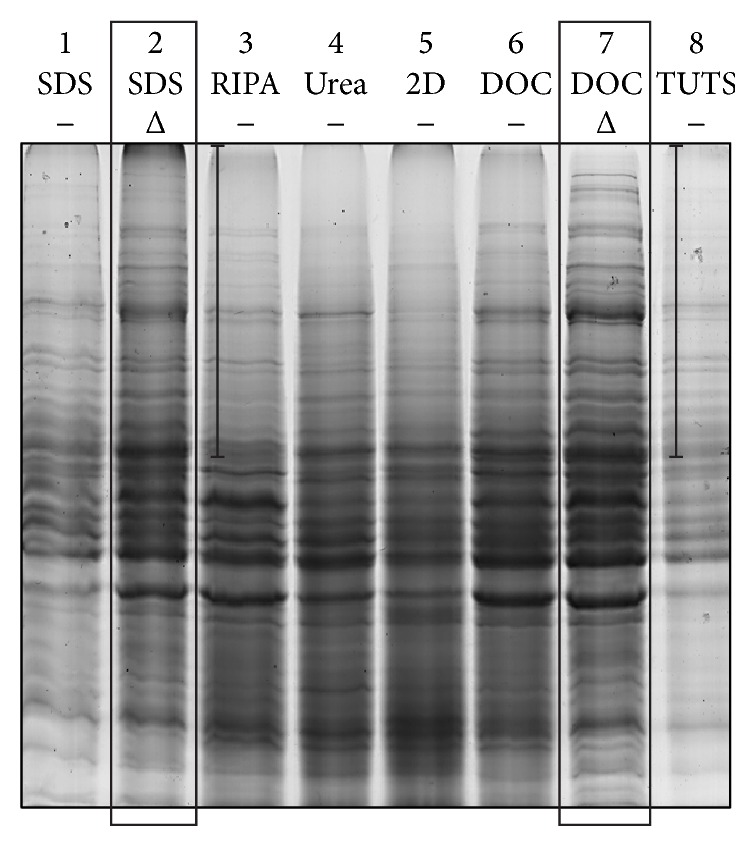
CBB stained gel from the 1D SDS-PAGE of proteins extracted from liver tumor samples using various extraction buffers. The loading concentration of each sample reflects the amount of proteins extracted from the liver samples before trypsin digestion. Larger number of protein bands would mean larger number of proteins extracted. Darker protein bands from each lane would mean a higher amount of proteins extracted. Black boxes indicate the two best extraction buffers and conditions in terms of number of protein bands and the intensity of the CBB stain. The highlighted regions for Lanes 2 and 7 show a larger number of visible bands compared to other lanes. Δ: heat.

Following protein extraction from the harvested liver tumor tissue using the various extraction buffers, 1D SDS-PAGE analysis was performed to provide an initial visual indication of protein extraction efficiency. As shown in Figure 1, protein extraction using the DOC extraction buffer was potentially better than the other extraction buffers, as evident from the larger number of protein bands as well as higher intensity bands in the DOC-extracted protein lysate samples. Our results were comparable to a previous study conducted by Proc et al. [23], who demonstrated that both SDS and DOC were more superior denaturants than urea in terms of the greater amount of solubilized human plasma proteins. This could explain the larger number of protein bands in the liver tumor samples extracted with SDS or DOC. However, in extraction buffers like RIPA and TUTS, the concentration of SDS and DOC could be too low to obtain comparable results with those of SDS or DOC extraction buffers.

Furthermore, as shown in Figure 1, the addition of the heat-denaturing step in our protocol greatly increased the extraction efficiency in SDS-extracted as well as DOC-extracted samples. In contrast, the introduction of the heat denaturing step by Proc et al. [23] did not show a significant increase in the digestion efficiency of human plasma proteins. Our observations were based on the amount of proteins extracted directly from a tissue rather than the digestion efficiency of trypsin investigated by the authors. Additionally, our results were based on the whole liver tumor tissue rather than just plasma proteins. Hence, the addition of heat denaturation step could greatly improve the amount of proteins extracted in our study. Hence, our results indicated the need to include the heat denaturation step to improve the protein extraction efficiency from the whole tissue.

### 3.2. 1D LC-MS/MS Shotgun Analysis of SDS- and DOC-Extracted Liver Tumor Samples

**Figure 2:**
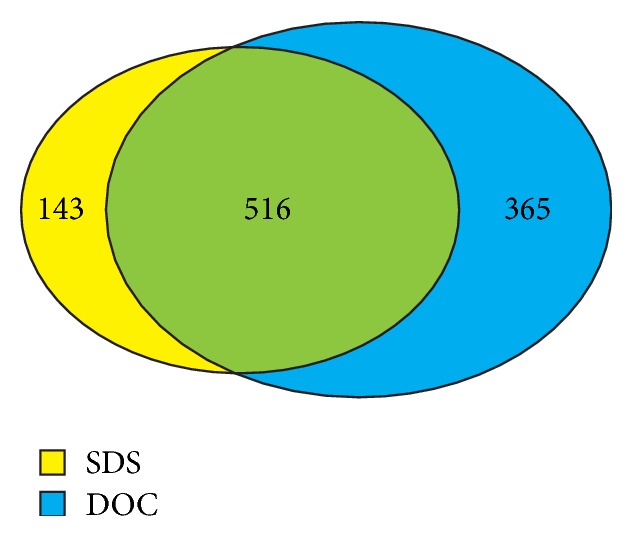
A comparison between the identified proteins from SDS-heat- and DOC-heat-extracted samples. A total of 1,024 unique proteins were identified from 1D shotgun analysis.

Following the above observations, both SDS-heat-extracted (SDSΔX) and DOC-heat-extracted (DOCΔX) samples were subjected to 1D LC-MS/MS shotgun proteomics (1D shotgun) analysis to determine the number of proteins that could be identified. In our 1D shotgun results, 659 and 881 unique proteins were identified from SDSΔX and DOCΔX samples, respectively (Supplementary Tables  1 and 2) (see Supplementary Material available online at http://dx.doi.org/10.1155/2015/763969). Between the two sets of identified proteins, there were 516 overlapping proteins, with 143 and 365 proteins uniquely found in SDSΔX and DOCΔX samples, respectively (Figure 2). Therefore, the use of DOC-heat might be more effective than SDS-heat in extracting liver proteins or DOC-heat might have improved the downstream sample processing mainly including trypsin digestion and MS analysis.

Although no direct comparison of the 1D shotgun profile between SDSΔX and DOCΔX liver tumor tissue samples has been reported in literature, a study by Zhou et al. [24] on the evaluation of the application of SDS in the proteomic analysis of rat hippocampal plasma membrane has shown that the use of DOC has resulted in a larger, although insignificant, number of total identified plasma membrane proteins or membrane-associated proteins than the SDS method. Our results also showed a difference in the total number of proteins identified between SDSΔX and DOCΔX samples, with the latter having a significantly larger number of proteins identified.

**Figure 3:**
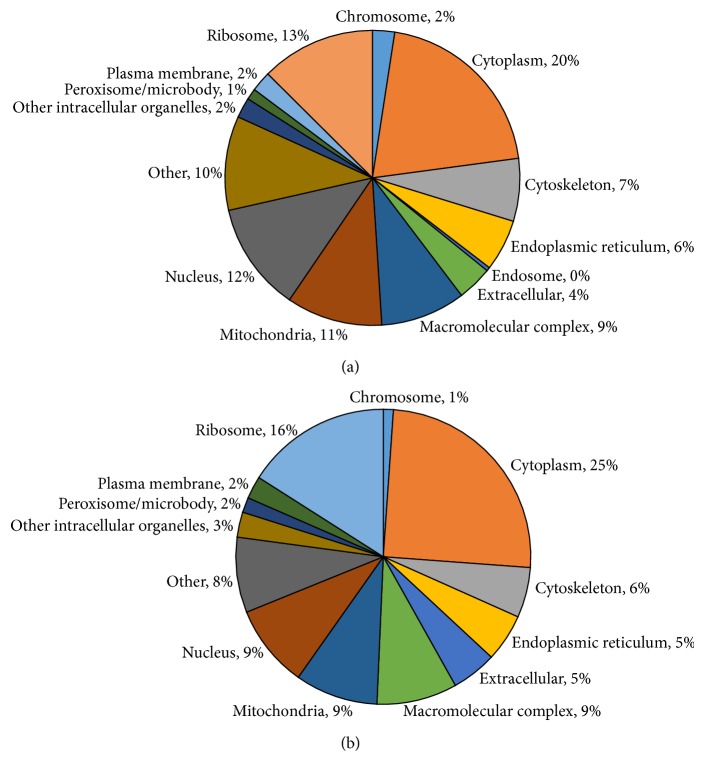
Subcellular localization of the identified proteins based on GO analysis. (a) 881 proteins identified in DOCΔX samples; (b) 659 proteins identified in SDSΔX samples generated using STRAP. The identified subcellular localization profiles are largely similar between both samples, with only the DOCΔX samples having proteins located in the endosome. The larger number of proteins identified from the DOCΔX samples also showed more even distribution of proteins across all subcellular locations. Percentages are rounded to the nearest whole number.

To determine whether different proteins identified from the two groups differ in terms of their subcellular localization, we conducted a GO analysis of the proteins using the* D. rerio* GOSLIM database. In the GO analysis of SDSΔX and DOCΔX samples, majority of the proteins are found in the cytoplasm (20% and 25% resp.; Figure 3). The subcellular localization profiles of both samples are observed to be very similar, but the additional proteins identified from the DOCΔX samples resulted in more even distribution of protein in the various subcellular locations, even detecting proteins in the endosome, which is absent from the SDSΔX sample. Our results highlighted the comparability of DOC- and SDS-based extraction method in their proteome coverage.

### 3.3. 2D LC-MS/MS Shotgun Analysis of DOC-Extracted Liver Tumor Sample

Since the DOC extraction buffer was able to extract more proteins from the nucleus and various organelles compared to the SDS extraction buffer, this could potentially increase the proteome coverage of the whole zebrafish liver using the DOC extraction buffer. To further increase the coverage of the whole proteome for the liver tumors, we conducted a 2D LC-MS/MS shotgun (2D shotgun) analysis on the DOCΔX sample since our 1D shotgun analysis revealed a better proteome coverage using DOC extraction buffer.

By 2D shotgun analysis, we identified a total of 4,790 unique proteins from the DOCΔX sample. In comparison, a previous study conducted by Wang et al. [12] on the proteomic profiling of cytosolic component of the zebrafish liver has identified a total of 1,204 proteins via the combination of three extraction methods as compared to a single one in our study. In another study by Carlson et al. [11], the investigators have used 8 M urea buffer to extract the proteins from adult zebrafish liver tissue, identifying a total of 745 proteins. Furthermore, Abramsson et al. [10] have employed a mixture of chloroform and methanol to extract proteins from various adult zebrafish organs including the liver and they have identified a total of 1,394 proteins from multiple tissues including blood, brain, fin, heart, intestine, liver, and skeletal muscle.

**Figure 4:**
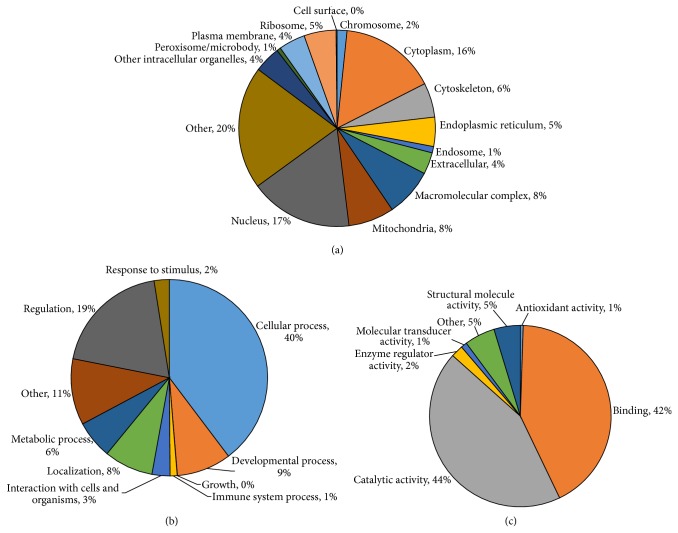
Distribution of proteins identified from the 2D shotgun dataset of DOC-extracted liver tumor samples based on (a) subcellular localization; (b) biological processes; (c) molecular functions. A total of 4,790 proteins were used in this GO analysis using STRAP. Percentages are rounded to the nearest whole number.

To the best of our knowledge, our study has by far the largest number of proteins reported to be identified from the zebrafish liver tissue. A full list of the proteins identified is presented in Supplementary Table  3. Using STRAP, the identified proteins were grouped according to their (1) subcellular locations, (2) biological processes, or (3) molecular functions. The data generated provide an overview of the proteins identified from* xmrk* oncogene induced zebrafish liver tumor (Figure 4).

The grouping of the 2D shotgun dataset indicated that the identified proteins were well represented across various subcellular locations, thus dismissing the possibility of subcellular location biasness. As illustrated in Figure 4(a), the identified proteins were originated from various subcellular locations, importantly from the nucleus (17%), mitochondria (8%), and the various organelles. In particular, the presence of plasma membrane proteins (4%) in our 2D shotgun dataset supports the previous study on the effectiveness of DOC in the extraction of poor water-soluble proteins like plasma membrane proteins by Zhou et al. [24]. This provides a further support in the use of DOC for tissue extraction of liver and other organs.

From our GO analysis for biological processes, we identified proteins involved mainly in cellular process (40%), regulation (19%), developmental process (9%), and localization (8%; Figure 4(b)). Our GO analysis for molecular functions identified proteins mainly with catalytic activity (44%) and binding functions (42%; Figure 4(c)). From our GO analysis, we have demonstrated the capability of our DOC-based protein extraction method to generate proteomic data, providing a platform to allow protein extraction from various organs for the study of diseases via proteomic approaches.

### 3.4. Identification of Proteins Involved in Important Signaling Pathways of Liver Cancer

Since the protein samples for our proteomic analysis were derived from liver tumors induced by expression of the* xmrk* oncogene, it is interesting to see if our DOC-based extraction method was able to identify proteins from pathways related to liver cancer. Our IPA results identified proteins involved in various diseases and biological functions such as cancer (2545 proteins), cell cycle progression (268 proteins), cell death (938 proteins), and proliferation of cells (1009 proteins) which could provide us with insight into liver cancer in future studies. More pieces of detailed information pertaining to the proteins classified into the respective diseases and biological functions with significance to liver cancer were provided in Supplementary Table  4.

**Figure 5:**
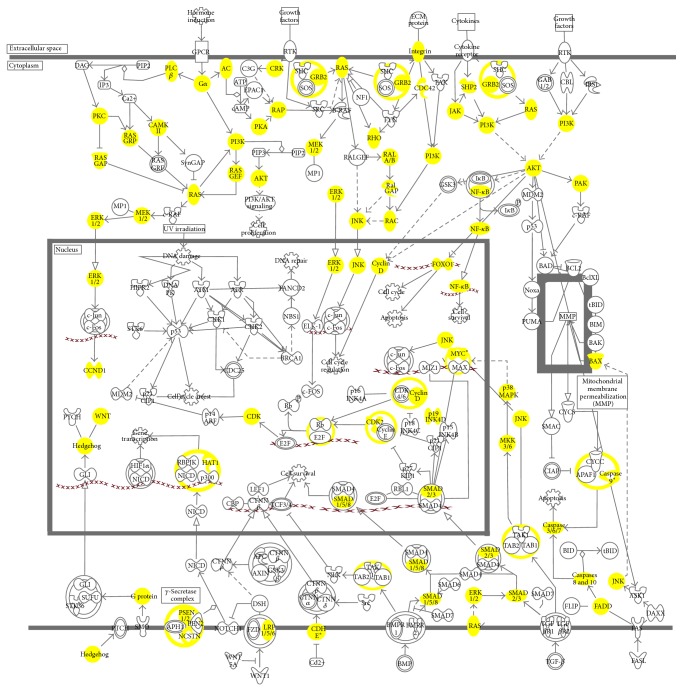
The major pathways involved in the molecular mechanisms of cancer as adapted from Ingenuity Pathway Analysis (IPA) database. The highlighted proteins in yellow depict our identified proteins from our 2D-LC-MS/MS analysis. A total of 77 proteins from our identified dataset from our 2D-LC-MS/MS are associated with the molecular mechanisms of cancer.

Further analysis using IPA revealed high coverage of our identified proteins in numerous canonical pathways. A close look into the pathways involved in the molecular mechanism of cancer identified a total of 77 associated proteins from our dataset, and Figure 5 shows the coverage of these proteins in the various cancer pathways. The coverage is relatively extensive, with many proteins identified upstream of pathways such as EGFR-Ras-mitogen-activated protein kinase (MAPK) and phosphoinositide 3-kinase/protein kinase B (PI3K/AKT). The carcinogenesis of liver cancer consists of a complex, multifactorial, stepwise development [25]. These include genetic mutations affecting signaling pathway such as Wnt-*β*-catenin, hedgehog, hepatocyte growth factor/mesenchymal-epithelial transition factor (HGF/c-Met), insulin-like growth factor (IGF), PI3K/AKT/mammalian target of rapamycin (mTOR), MAPK, p53, phosphoretinoblastoma (pRb), Janus kinase-signal transducers and activators of transcription (JAK-STAT), and transforming growth factor-*β* (TGF*β*) pathways [25–27].

These genetic alternations will result in changes to the cellular proteome; thus the use of comprehensive approaches to profile for these changes might provide insights to the molecular mechanism leading to the development of liver cancer. Our IPA results revealed good representation of our identified proteins in Wnt-*β*-catenin (28 proteins), HGF/c-Met (28 proteins), IGF (32 proteins), PI3K/AKT/mTOR (79 proteins), MAPK (51 proteins), JAK-STAT (21 proteins), and TGF*β* (21 proteins) pathways. A detailed list of the identified proteins for each pathway is listed in Supplementary Table  4. These results again demonstrated the potential of our DOC-based protein extraction method in high-throughput proteomic studies to elucidate the molecular progression of diseases.

**Figure 6:**
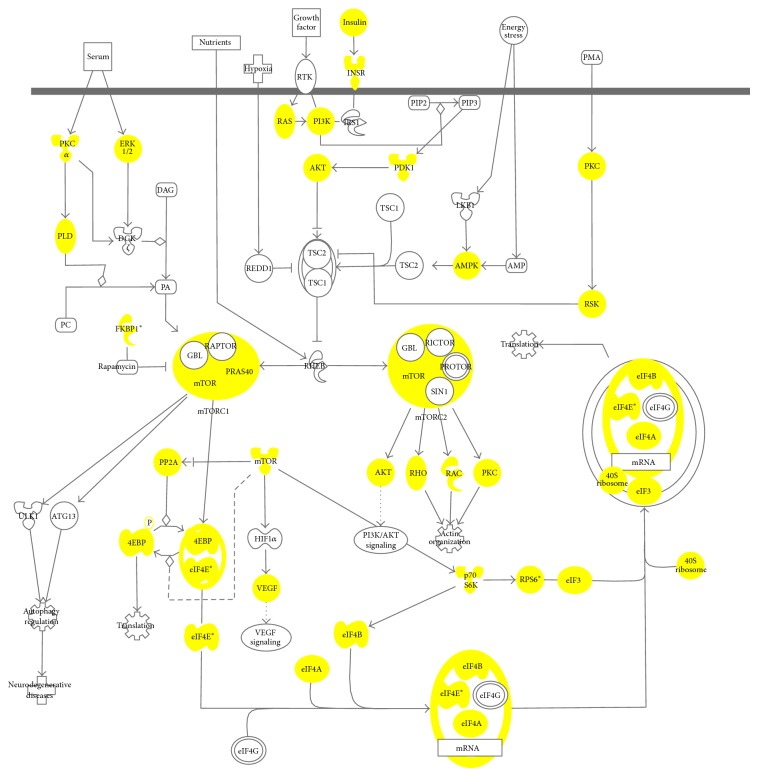
The PI3K/AKT/mTOR pathway as adapted from Ingenuity Pathway Analysis (IPA) database. The highlighted proteins in yellow depict our identified proteins from our 2D-LC-MS/MS analysis. A total of 79 proteins from our identified dataset from our 2D-LC-MS/MS are associated with this pathway.

Two of the fundamental hallmarks of cancers are the ability to sustain prolonged cell proliferation and the use of abnormal metabolic pathways to generate energy. The PI3K/AKT/mTOR pathway is documented to regulate cell growth, aging, and metabolism [28]. Furthermore, our previous study also identified the presence of dysregulated PI3K/AKT/mTOR pathway in* xmrk* zebrafish liver tumors [29]. It has been documented that this pathway is upregulated in 40–50% of hepatocellular carcinoma, which is the most common primary cancer of the liver [30–32]. Henceforth, we subjected our 2D shotgun dataset to pathway analysis using IPA. Our results showed that 79 associated proteins were identified belonging to the PI3K/AKT/mTOR pathway, notably Ras, PI3K, AKT, mTOR, p70S6K, 4EBP, and eIF4E proteins (Figure 6). With such a high coverage of proteins associated with the PI3K/AKT/mTOR pathway, it would be highly beneficial for further studies pertaining to this pathway in relation to liver cancer. This again demonstrates the potential of our DOC-based protein extraction method in high-throughput proteomic studies to elucidate the molecular progression of diseases.

### 3.5. Overview of the Strengths of DOC in Protein Extraction from Tissue Samples

**Figure 7:**
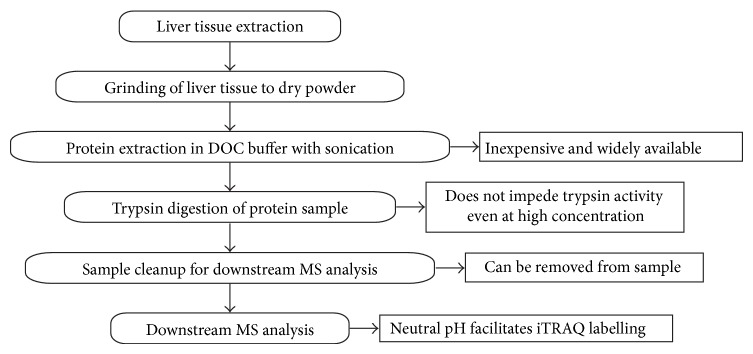
An overview of advantages of DOC as a protein extraction buffer for proteomic analysis.

Our study demonstrated the feasibility of using DOC in the extraction of proteins from zebrafish liver tumor tissue.  Figure 7 presents a general overview of the advantages of using DOC as an extraction buffer for proteomic studies. DOC is an inexpensive and widely available denaturant. In addition, its acid-insolubility and precipitation at low pH enable its removal from the sample before LC-MS/MS analysis [24]. This is one of the key features over the more commonly used SDS. SDS is impossible to be removed by reversed-phase high performance LC, and trace amount of SDS (<0.01%) will disrupt the separation of peptides with LC [33, 34]. Moreover, SDS suppresses the ionization in matrix-assisted laser desorption/ionization and electrospray ionization MS approaches [23], causing the loss of signal that could potentially result in the low-confidence detection of proteins.

Another key to obtain a large coverage of proteins in a proteome profiling study is the complete digestion of the proteins into peptides. Thus, any chemical or solvent that impedes the activity of the enzyme (commonly trypsin) used would result in reduced enzymatic digestion and peptide numbers. The reduced amount of peptides will result in reduced detection, and hence the identification of the protein will be affected. However, the activity of trypsin is largely unaffected by high concentration of DOC solution, even up to 10% DOC [16]. This property allows the use of a higher concentration of the denaturant DOC to aid in solubilizing those hard-to-solubilize proteins.

Additionally, the pH value of 8 for DOC also facilitates trypsin digestion without the need to readjust the pH. Furthermore, it is also compatible with iTRAQ approach, which is by far the most commonly used quantitative proteomics profiling approach. The pH for iTRAQ labelling is optimally conducted at pH 8. Hence, the pH value does not need to be adjusted before iTRAQ labelling, reducing the amount of work needed as well as potential parallel sample processing variations. Interestingly, iTRAQ-based proteomic studies have a pH reduction step before applying the sample to SCX. Therefore, the reduction in pH could result in the precipitation of DOC, hence its removal.

The addition of heat during the processing of our DOCΔX sample further improved the protein extraction efficiency of DOC as highlighted in our previous session. Heat was not applied to extraction buffers containing urea because heat can break down urea to release isocyanate that can cause carbamylation of proteins [35]. In addition, heating of protein samples can induce protein aggregation without the presence of additives such as detergents [36]. Hence, heat could induce protein aggregation in the other lysis buffers containing the low concentration of detergents and denaturants. However, the high concentration of detergents in our SDS and DOC extraction buffers prevented potential protein aggregation with the addition of heat to our protein extraction step, further highlighting the strength of our DOC-heat-extraction method.

## 4. Conclusions

In conclusion, the advantages of DOC coupled with heat treatment have greatly increased the number of proteins identified by mass spectrometry in proteomic studies, and this further indicated the suitability and simplicity of DOC in protein extraction of tissue from liver and other organs. Our positive evaluation of DOC is in line with the study by Proc et al. [23], who have demonstrated the high digestion efficiencies, and the highest average reproducibility in the proteins detected among other chemicals and solvents, recommending DOC as the most ideal denaturant over SDS. More importantly, we have shown the suitability of DOC in protein extraction of complex tissue (liver tumors in this study) without compromising the quality and coverage of the proteome. This is further justified by our detection of low abundant proteins, thus allowing for the detection of potential cancer biomarkers in our liver tumor samples.

## Supplementary Material

The Supplementary material contains our identified protein list from our 1D shotgun analysis for both DOCΔX (Supplementary Table 1) and SDSΔX (Supplementary Table 2) samples.
Supplementary Table 3 contains our identified protein list from our 2D shotgun analysis for DOCΔX sample. Supplementary Table 4 contains the list of representative “canonical pathways” and “diseases and biological functions” with the respective proteins from our identified protein list from Supplementary Table 3 that we obtained from our pathway analysis using IPA.
Supplementary Data 1 contains the chromatograms from our 1D (both DOCΔX and SDSΔX) and 2D shotgun analysis (DOCΔX).

## Figures and Tables

**Table 1:** Overview of the various lysis buffer and the number of proteins identified using 1D LC-MS/MS shotgun analysis.

Number	Lysis buffer	Buffer component	Heat	Number of protein ID
1	SDS	1% SDS, 0.5 M TEAB	−	NA.
2	SDS	1% SDS, 0.5 M TEAB	+	622
3	RIPA	50 mM Tris, 150 mM NaCl, 1% NP-40, 1% DOC, and 0.1% SDS	−	NA.
4	Urea	9 M urea	−	NA.
5	2D	7 M urea, 2 M thiourea, 4% CHAPS, 20 mM DTT, and 40 mM Tris	−	NA.
6	DOC	5% DOC	−	NA.
7	DOC	5% DOC	+	823
8	TUTS	25 mM TEAB, 8 M urea, 2% triton X-100, and 0.1% SDS	−	NA.

TEAB: triethylammonium bicarbonate; TUTS: TEAB/urea/triton-X/SDS.
