# Supplementary material for: Dramatic Improvement of Proteomic Analysis of Zebrafish Liver Tumor by Effective Protein Extraction with Sodium Deoxycholate and Heat Denaturation
Source: Int J Anal Chem. 2015 Mar 19;2015:763969. doi: 10.1155/2015/763969 (PMC4383156; doi:10.1155/2015/763969)

Chromatogram of SDS buffer extracted zebra fish liver sample(1D-LC-MS)

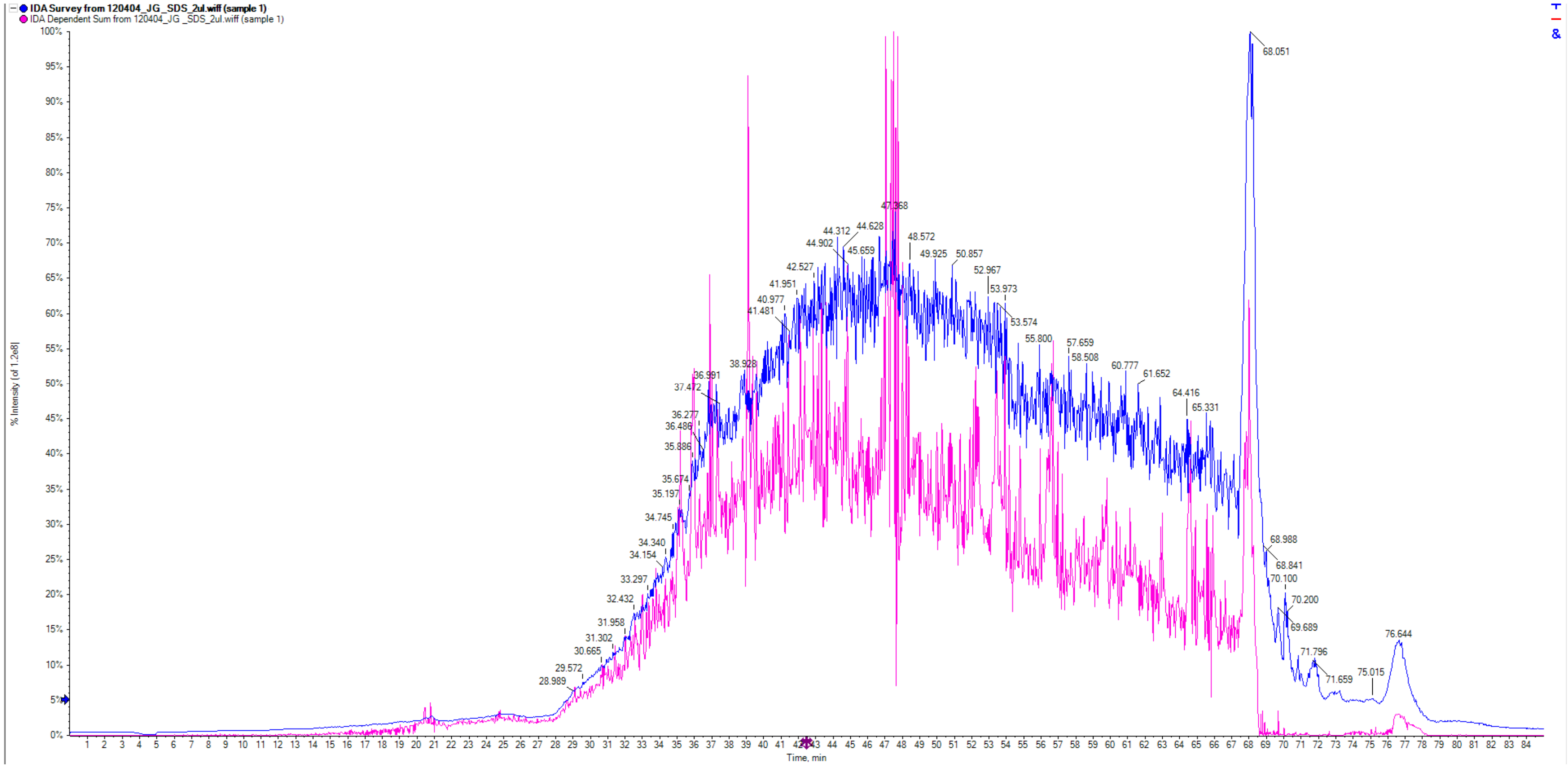

Chromatogram of DOC buffer extracted zebra fish liver sample (1D-LC-MS)

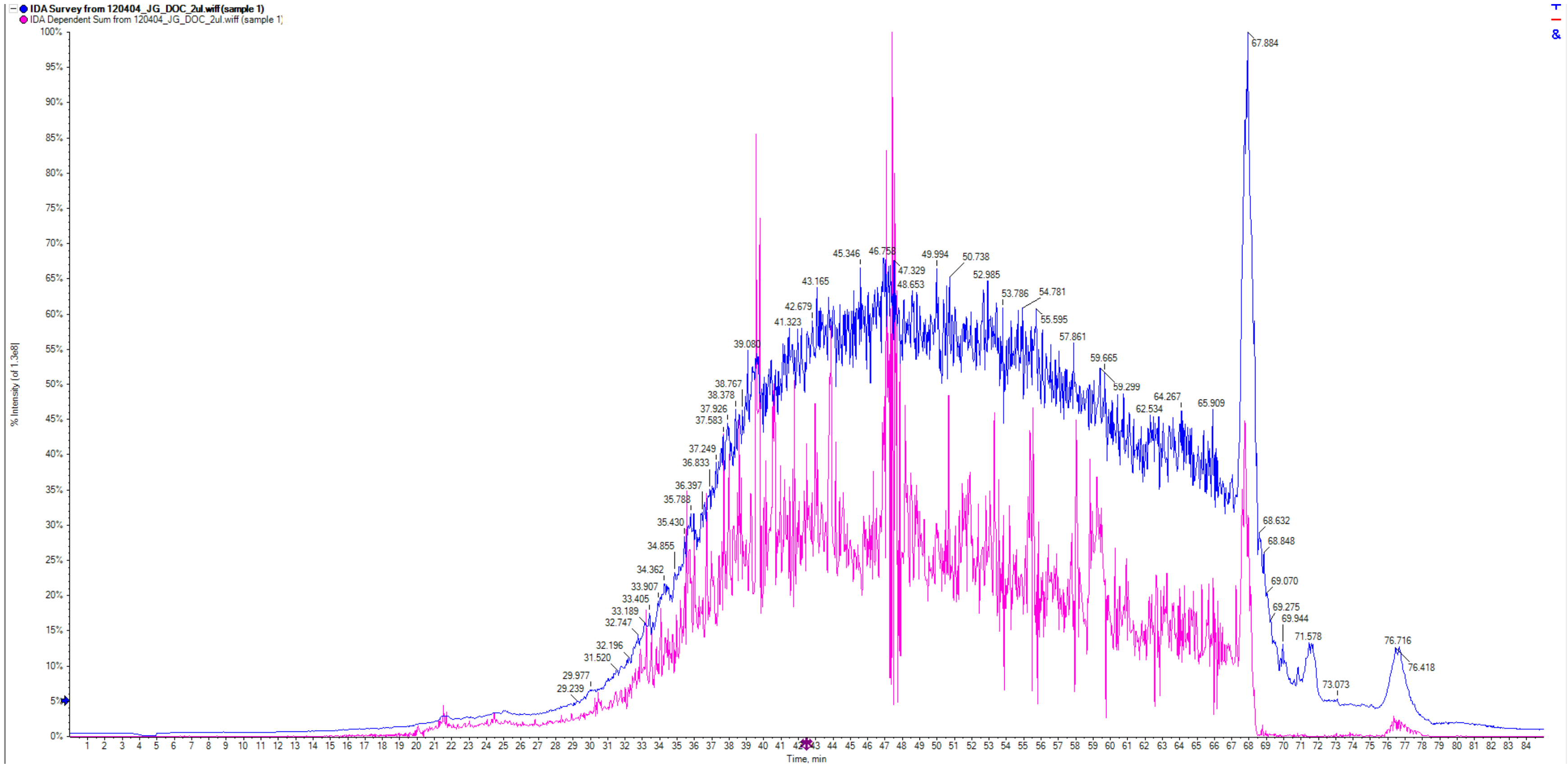

Chromatogram of DOC buffer extracted zebra fish liver sample_ All fraction overlay (2D-LC-MS)

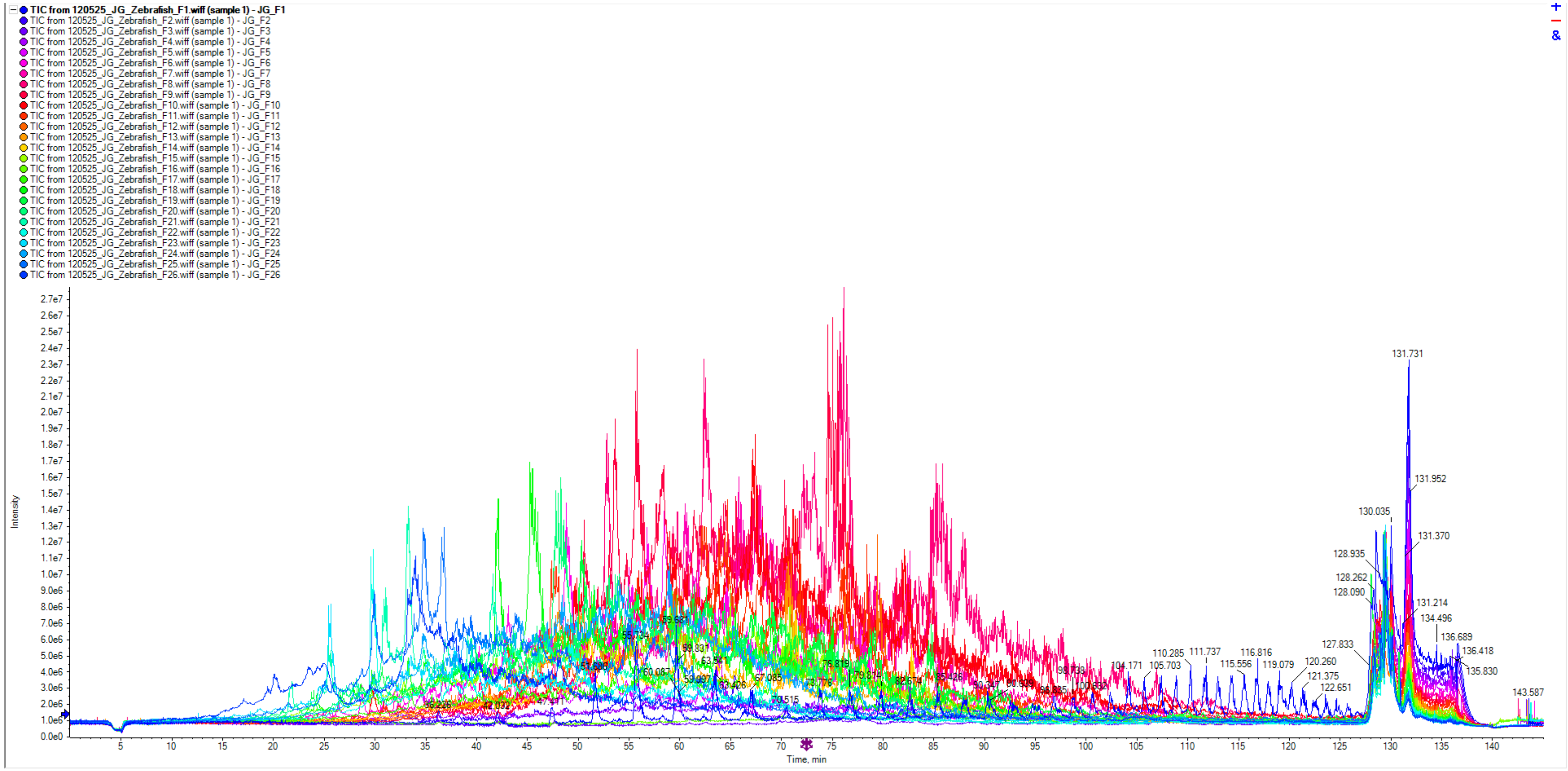

Chromatogram of DOC buffer extracted zebra fish liver sample_Fraction 1 (2D-LC-MS)

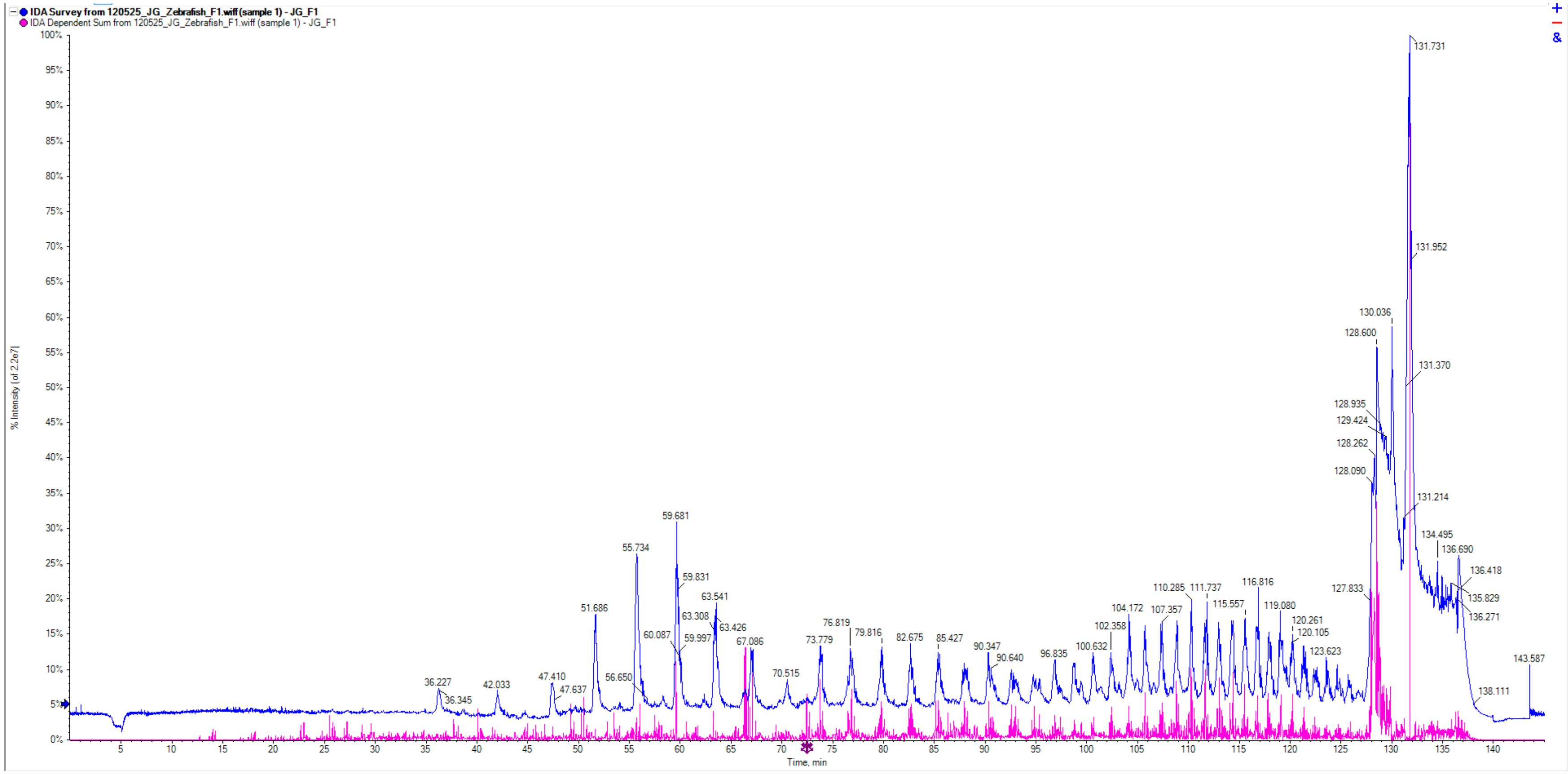

Chromatogram of DOC buffer extracted zebra fish liver sample_Fraction 2 (2D-LC-MS)

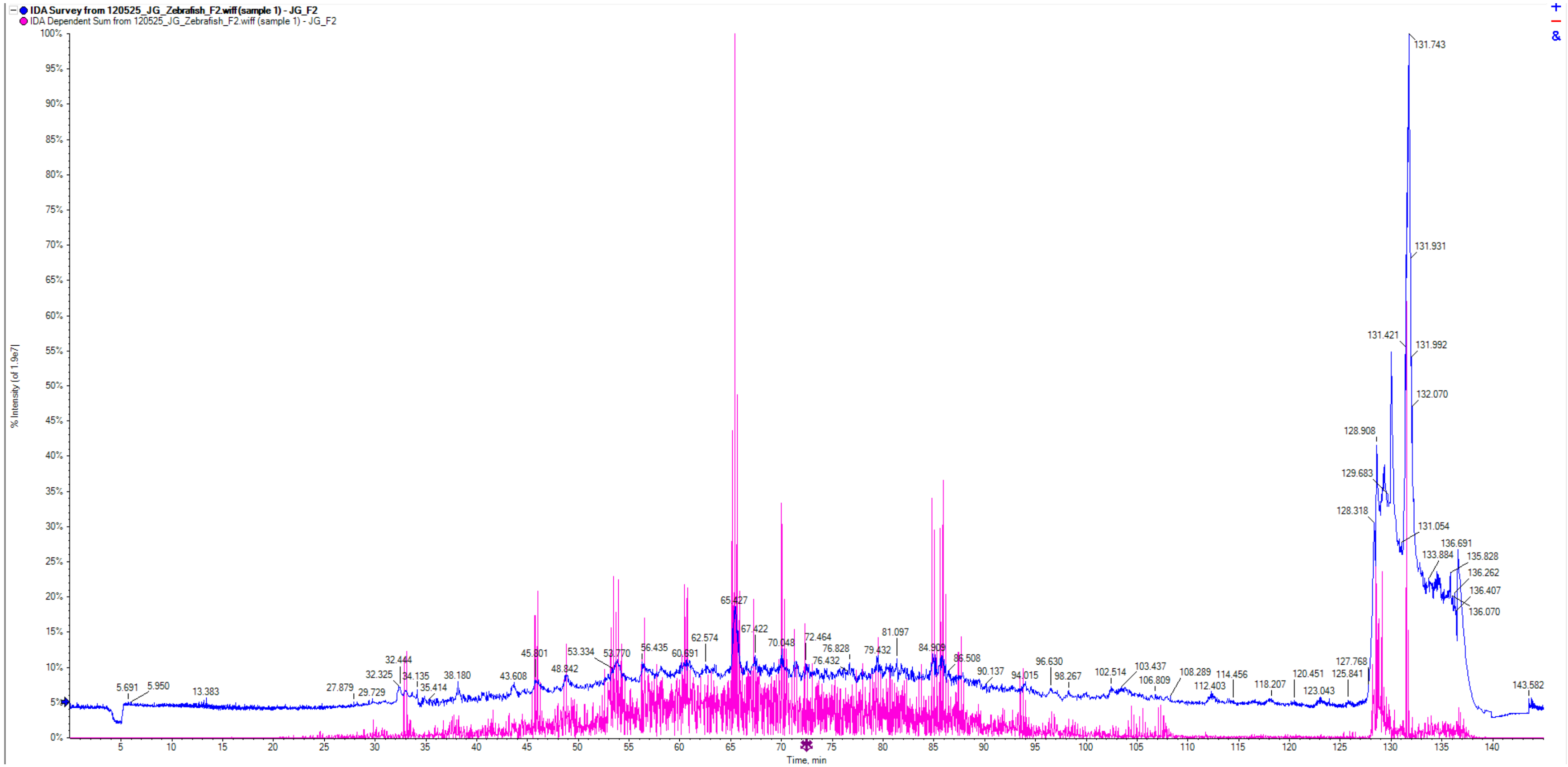

Chromatogram of DOC buffer extracted zebra fish liver sample_Fraction 3 (2D-LC-MS)

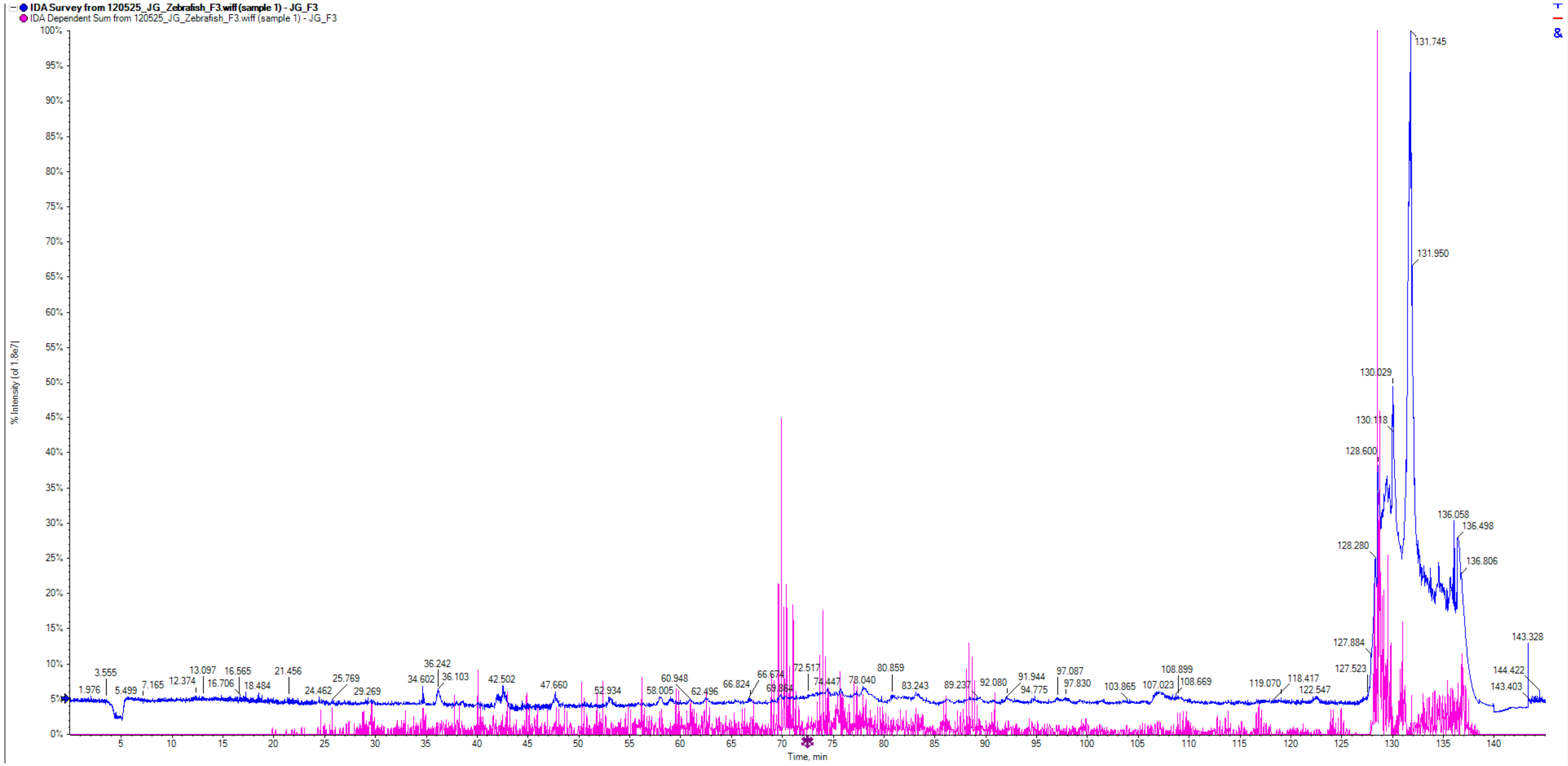

Chromatogram of DOC buffer extracted zebra fish liver sample_Fraction 4 (2D-LC-MS)

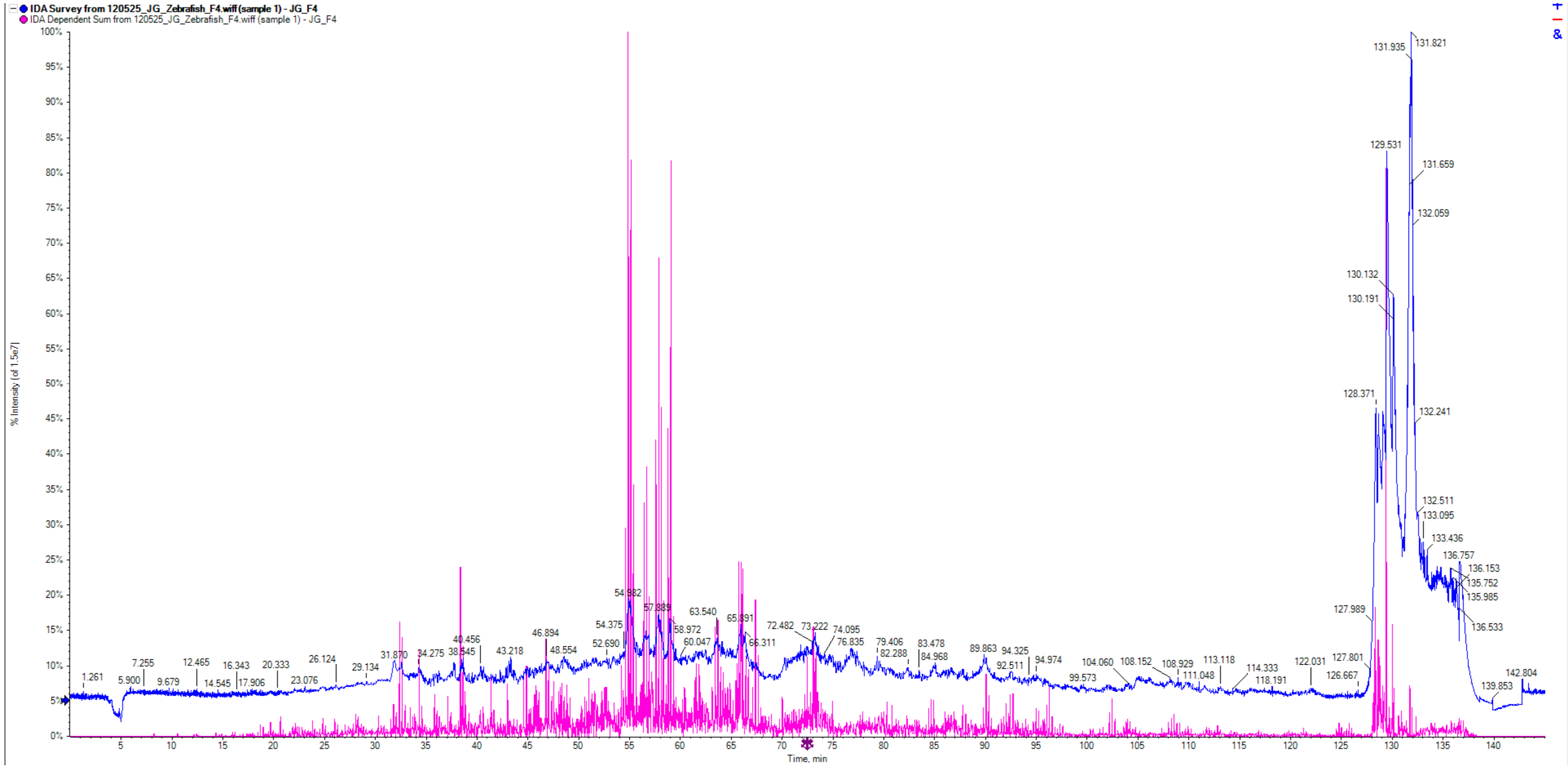

Chromatogram of DOC buffer extracted zebra fish liver sample_Fraction 5 (2D-LC-MS)

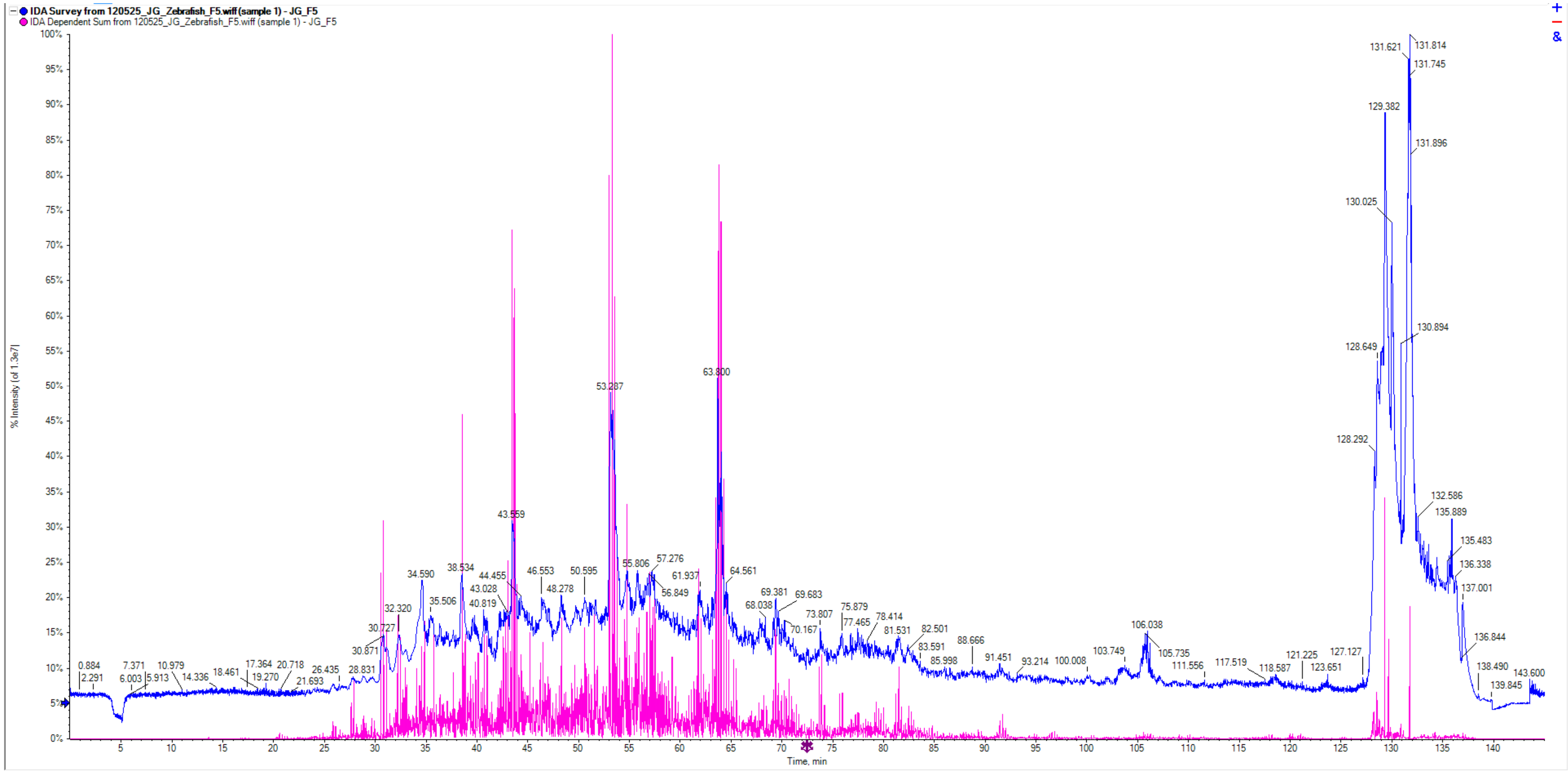

Chromatogram of DOC buffer extracted zebra fish liver sample_Fraction 6 (2D-LC-MS)

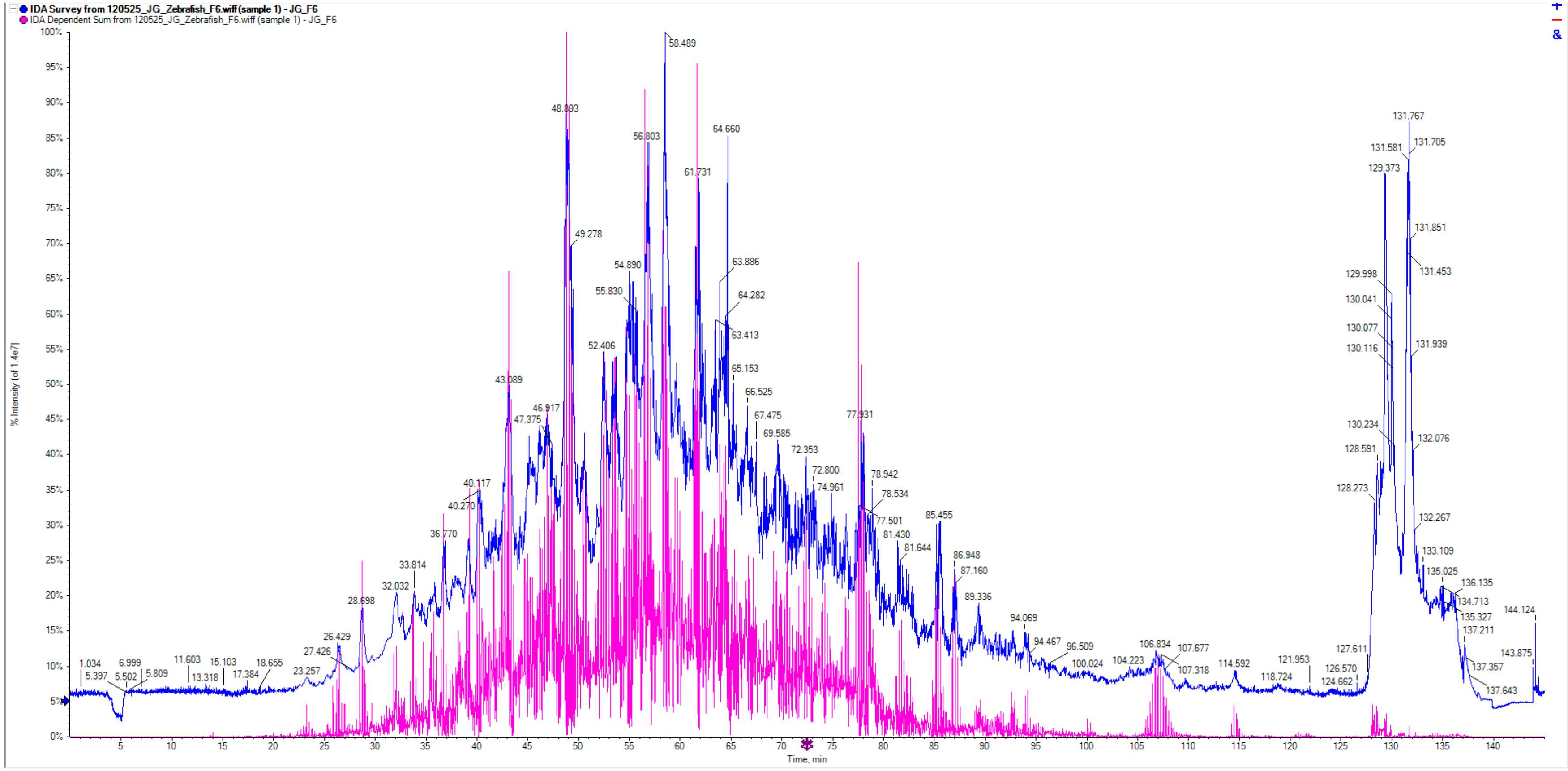

Chromatogram of DOC buffer extracted zebra fish liver sample_Fraction 7 (2D-LC-MS)

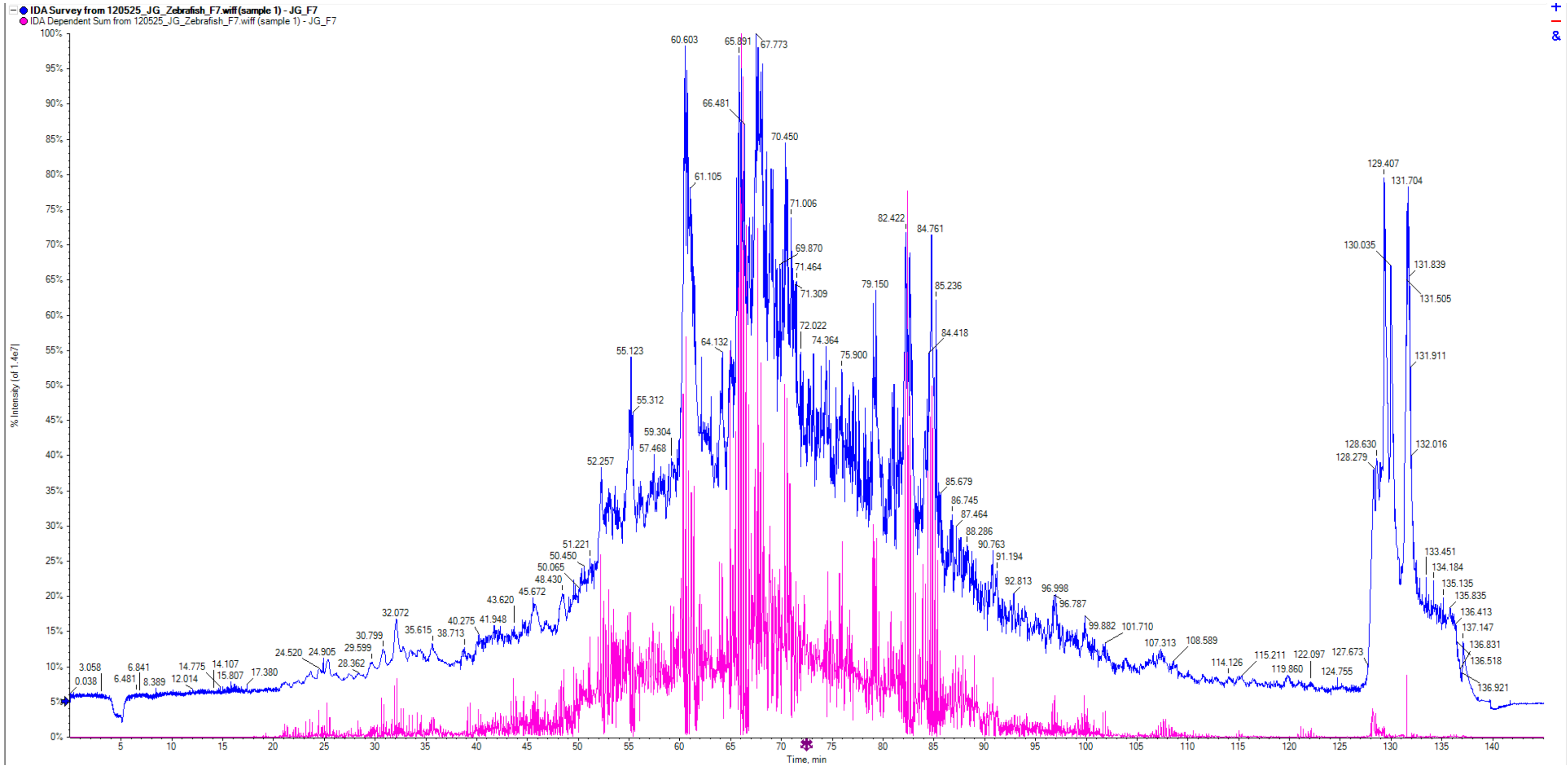

Chromatogram of DOC buffer extracted zebra fish liver sample_Fraction 8 (2D-LC-MS)

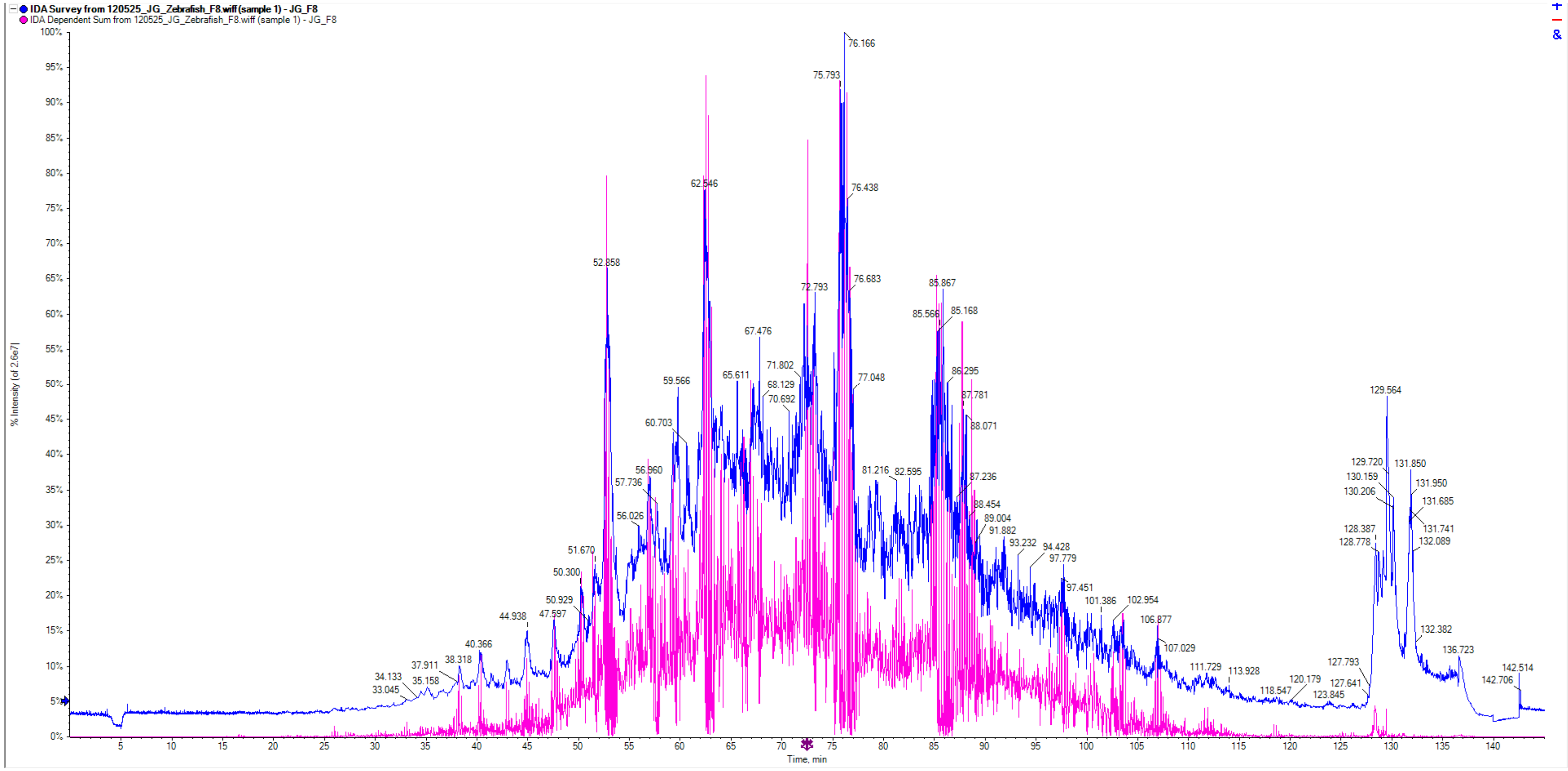

Chromatogram of DOC buffer extracted zebra fish liver sample_Fraction 9 (2D-LC-MS)

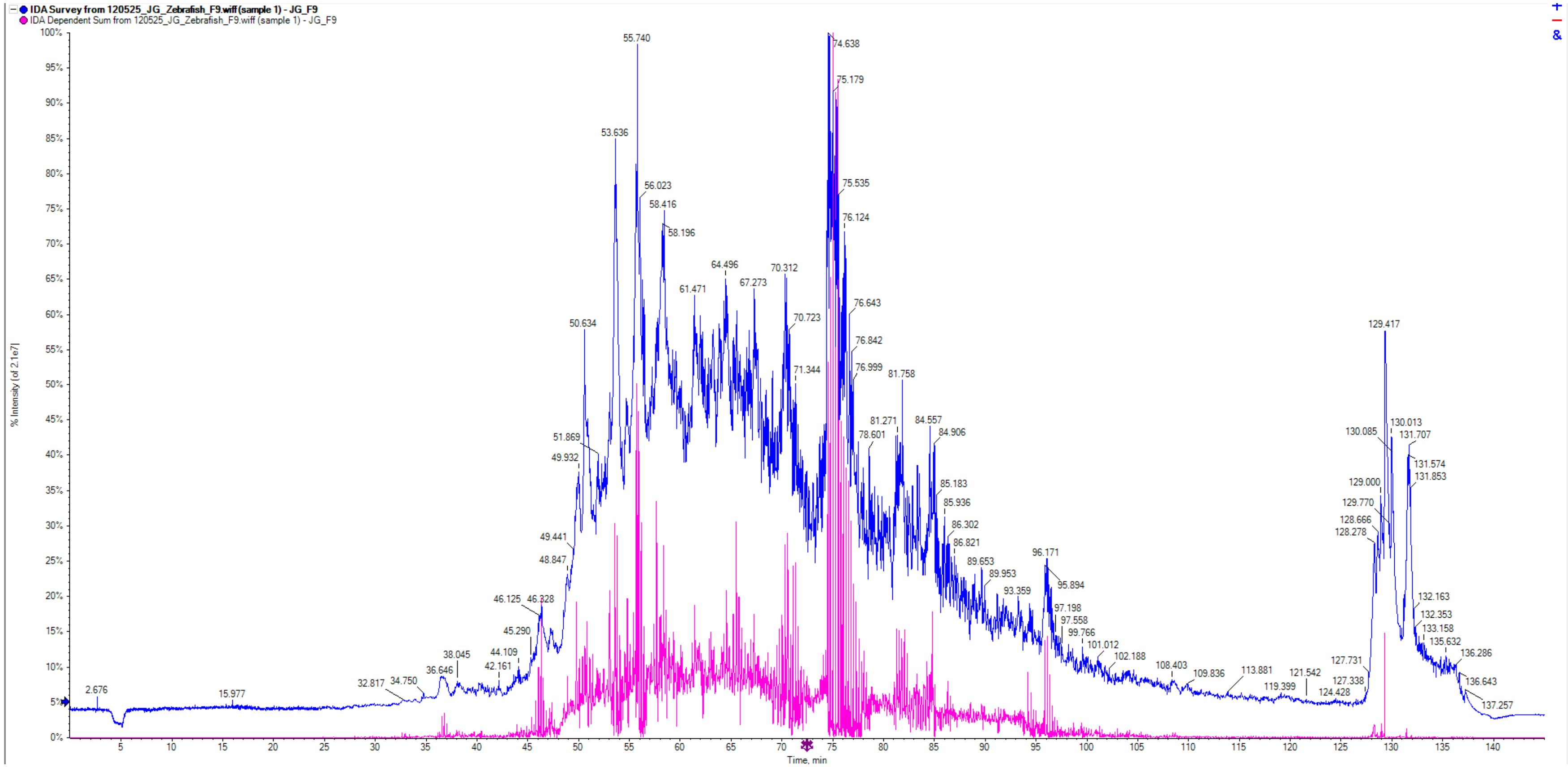

Chromatogram of DOC buffer extracted zebra fish liver sample_Fraction 10 (2D-LC-MS)

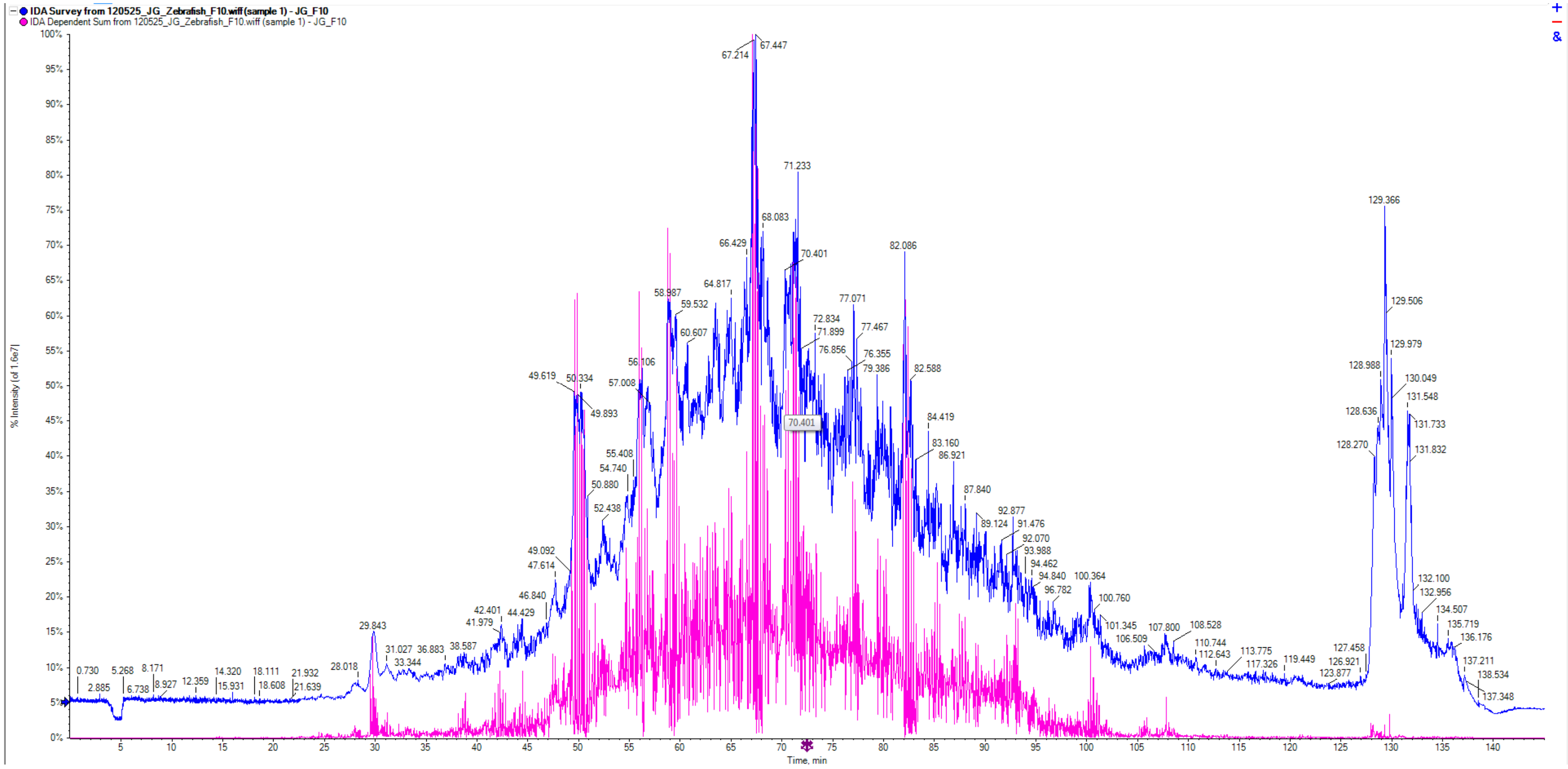

Chromatogram of DOC buffer extracted zebra fish liver sample_Fraction 11 (2D-LC-MS)

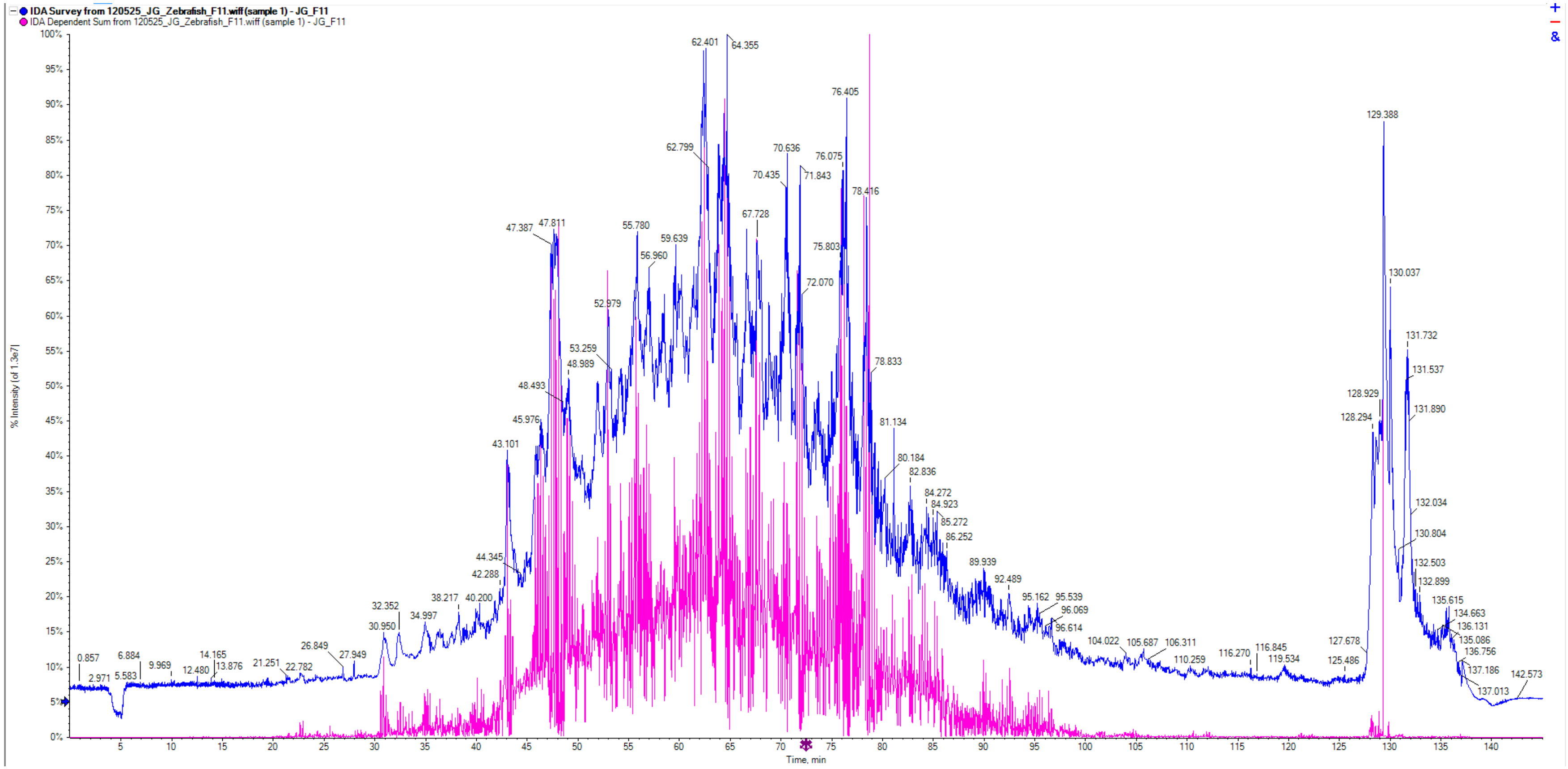

Chromatogram of DOC buffer extracted zebra fish liver sample_Fraction 12 (2D-LC-MS)

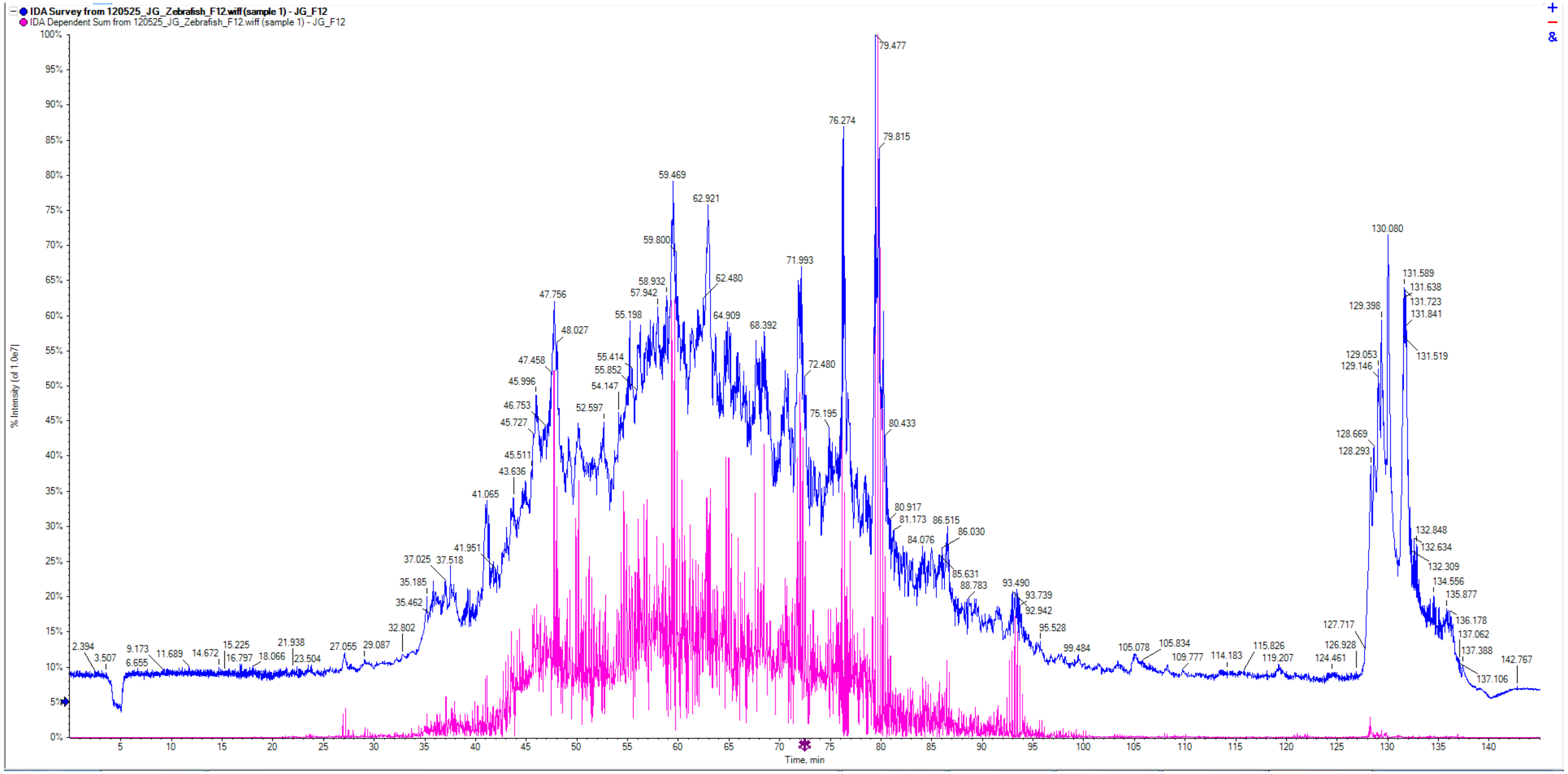

Chromatogram of DOC buffer extracted zebra fish liver sample_Fraction 13 (2D-LC-MS)

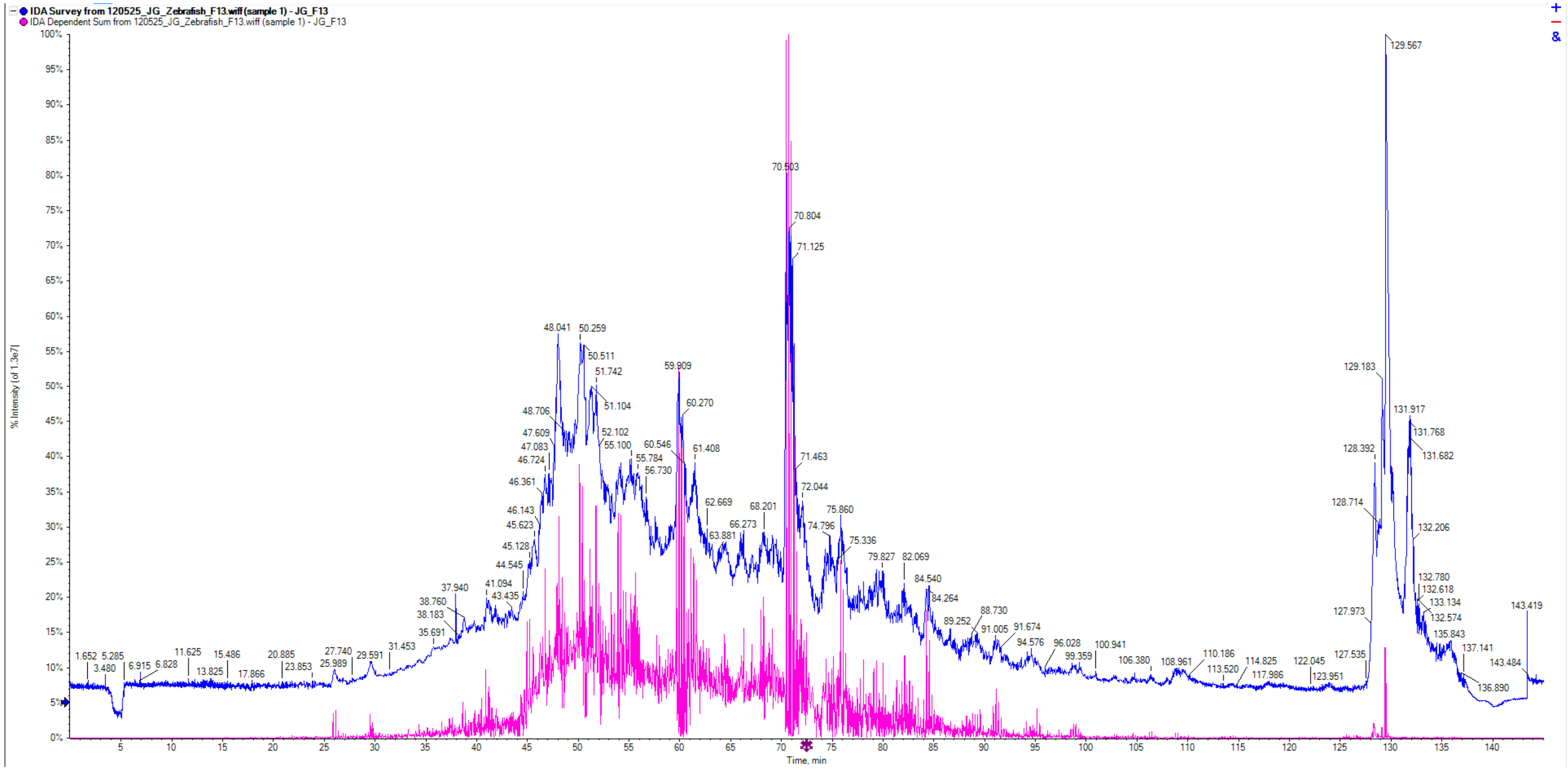

Chromatogram of DOC buffer extracted zebra fish liver sample_Fraction 14 (2D-LC-MS)

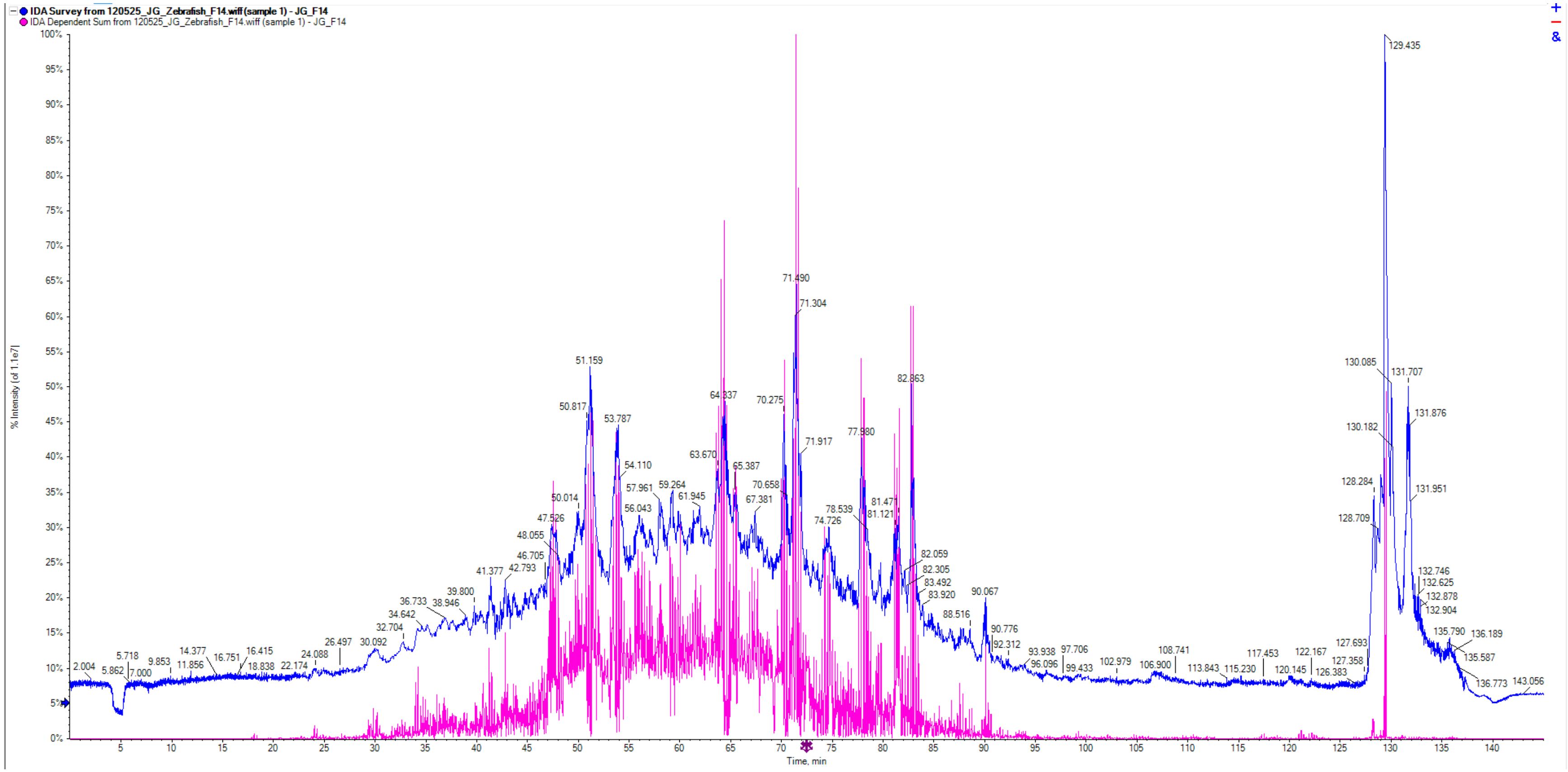

Chromatogram of DOC buffer extracted zebra fish liver sample_Fraction 15 (2D-LC-MS)

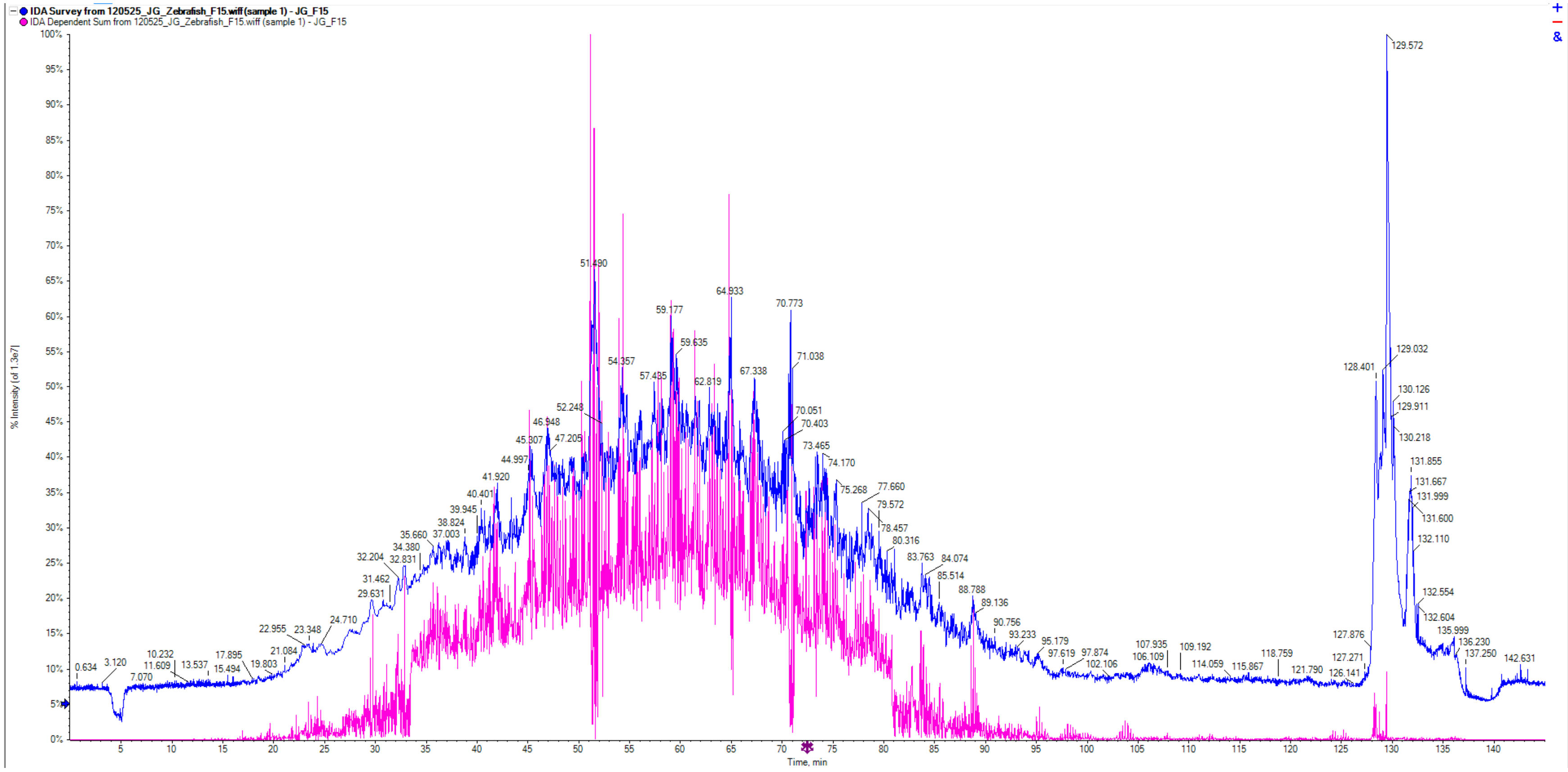

Chromatogram of DOC buffer extracted zebra fish liver sample_Fraction 16 (2D-LC-MS)

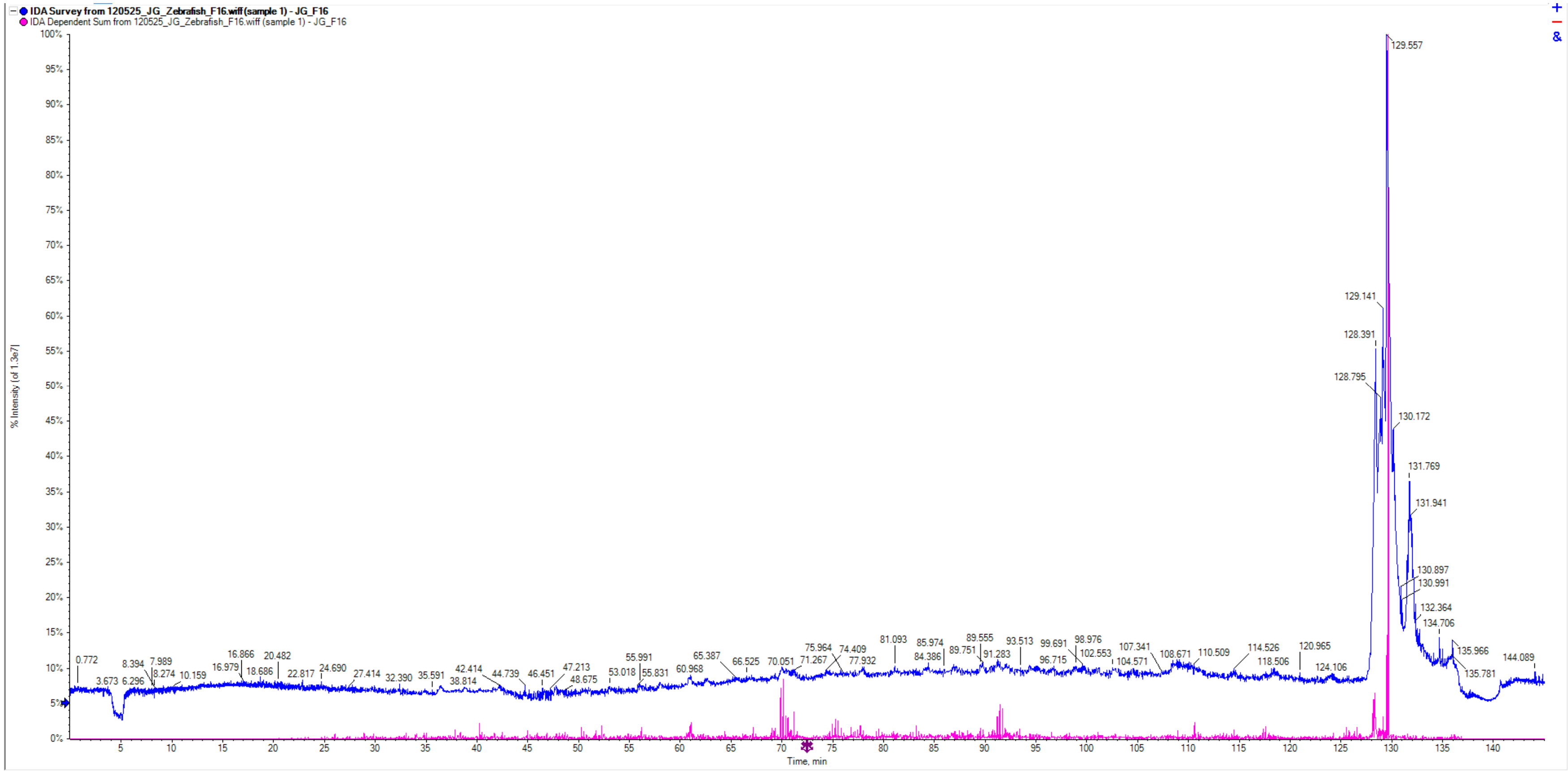

Chromatogram of DOC buffer extracted zebra fish liver sample_Fraction 17 (2D-LC-MS)

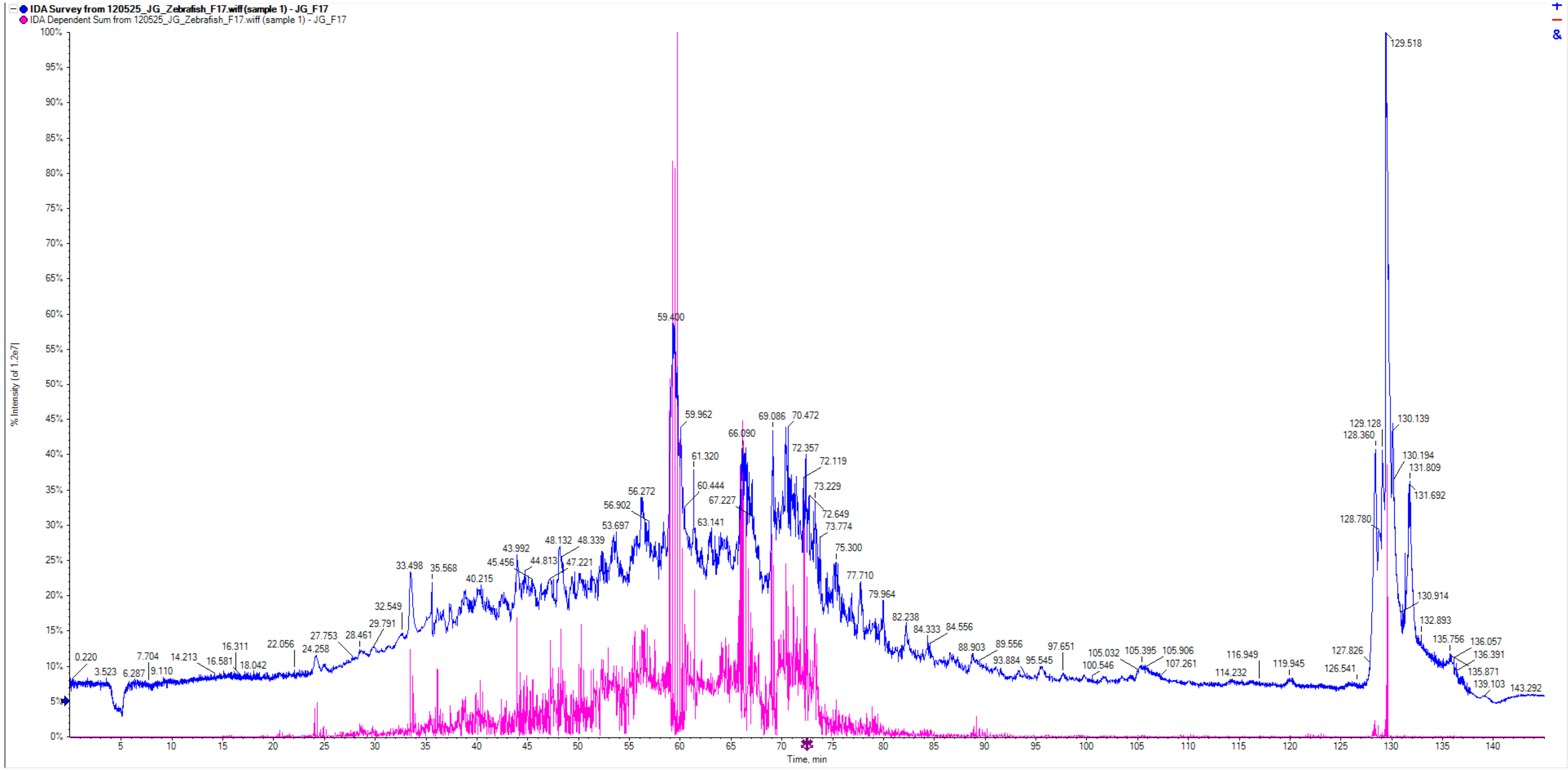

Chromatogram of DOC buffer extracted zebra fish liver sample_Fraction 18 (2D-LC-MS)

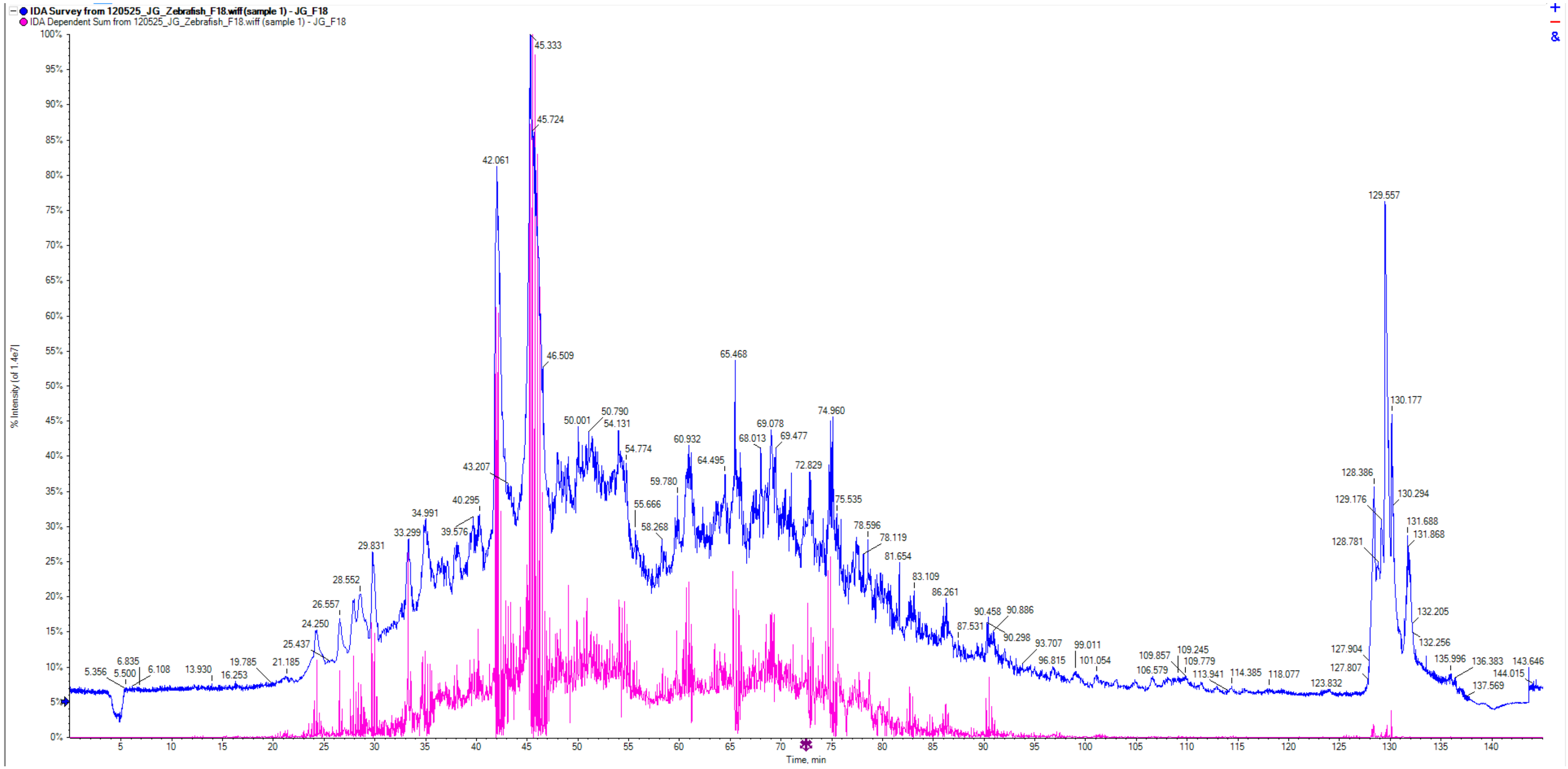

Chromatogram of DOC buffer extracted zebra fish liver sample_Fraction 19 (2D-LC-MS)

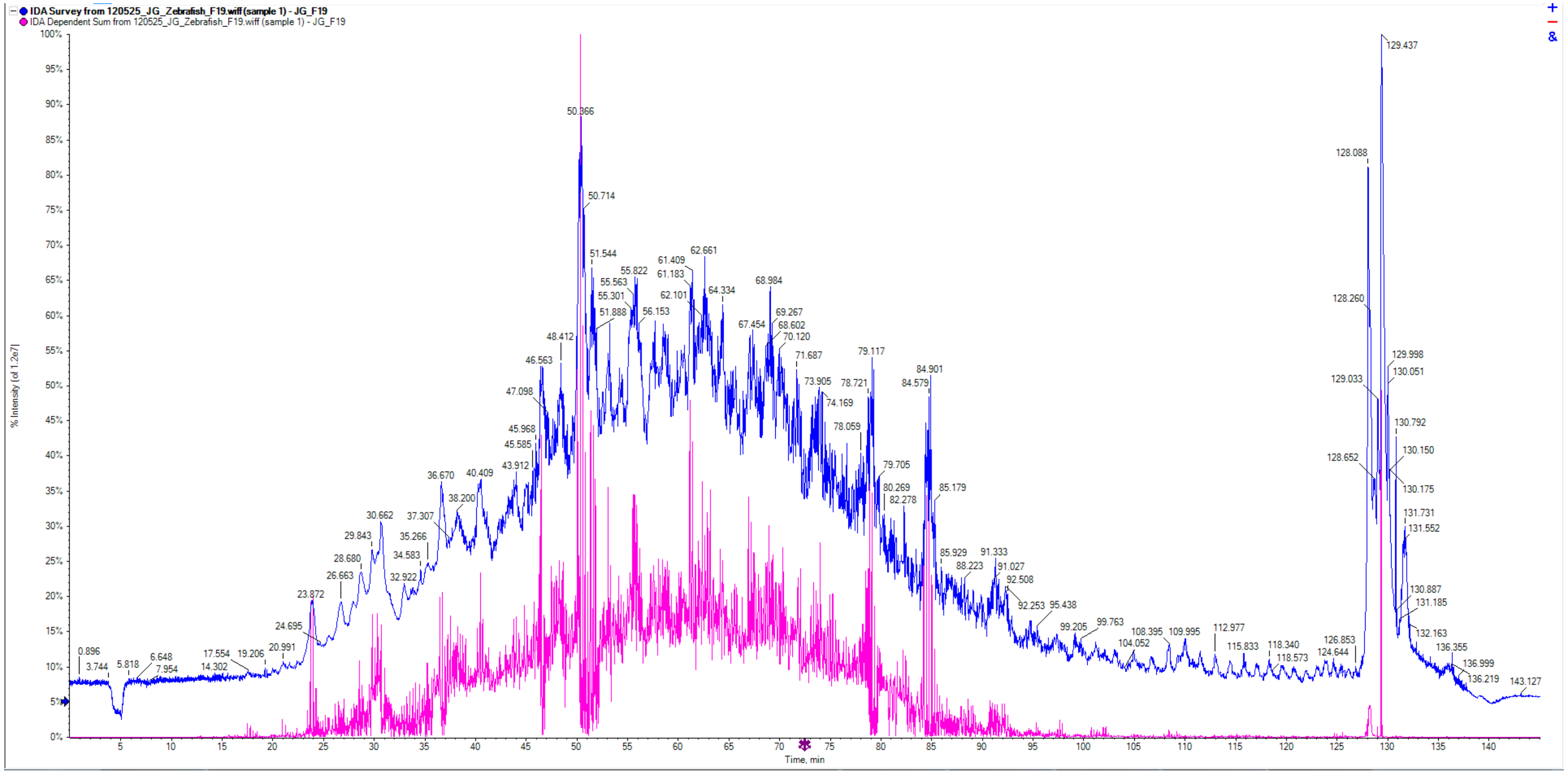

Chromatogram of DOC buffer extracted zebra fish liver sample_Fraction 20 (2D-LC-MS)

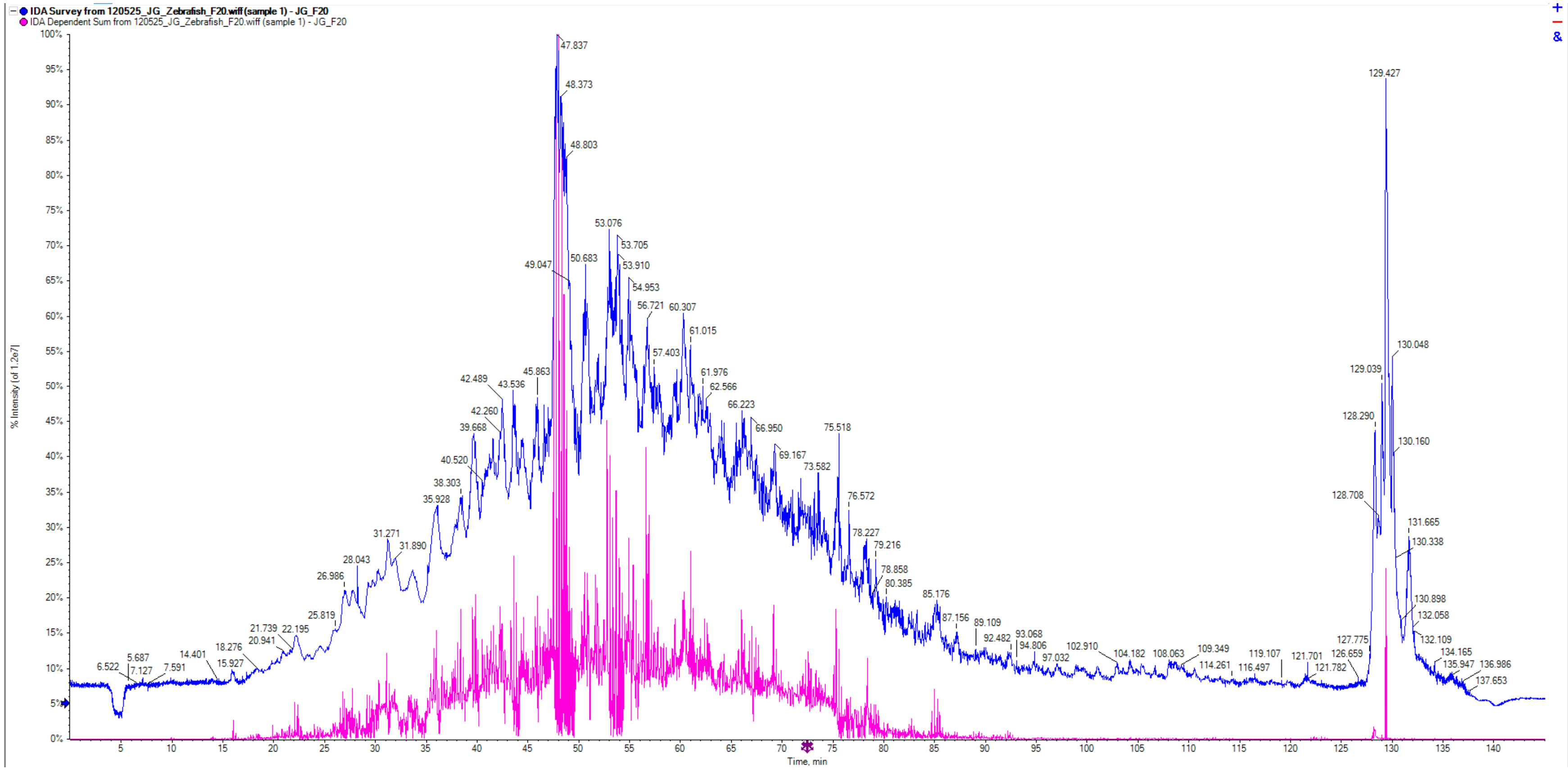

Chromatogram of DOC buffer extracted zebra fish liver sample_Fraction 21 (2D-LC-MS)

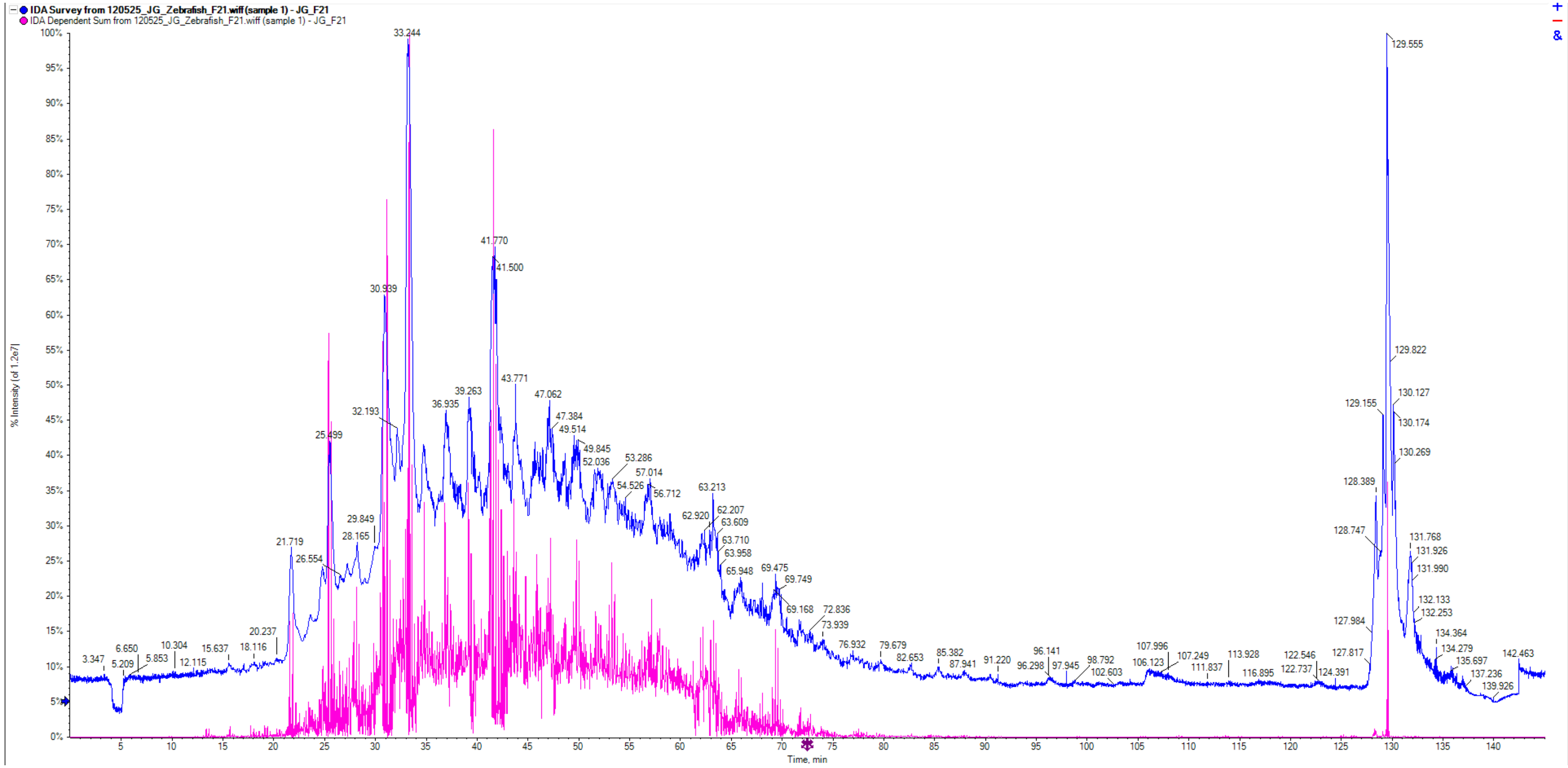

Chromatogram of DOC buffer extracted zebra fish liver sample_Fraction 22 (2D-LC-MS)

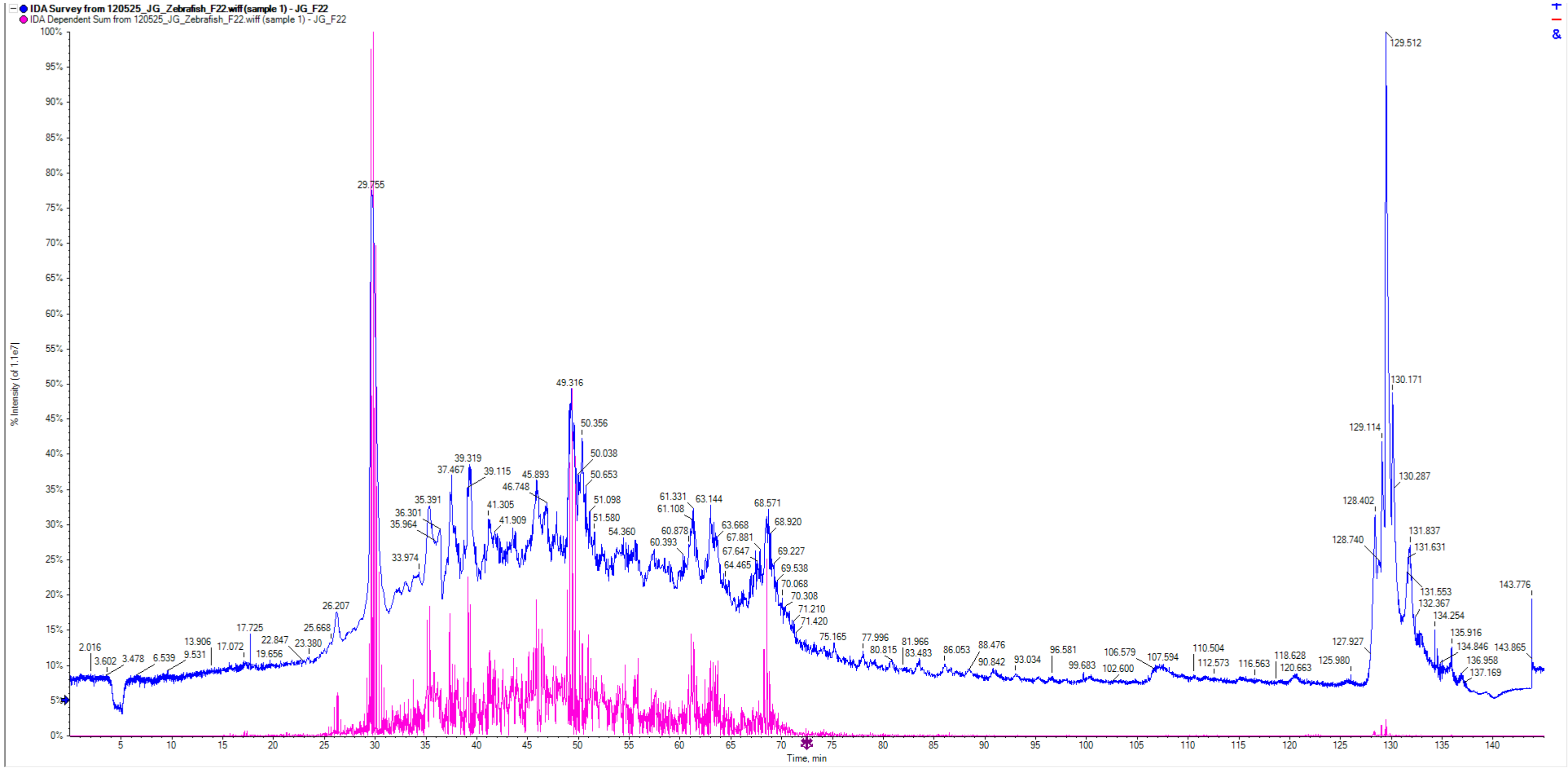

Chromatogram of DOC buffer extracted zebra fish liver sample_Fraction 23 (2D-LC-MS)

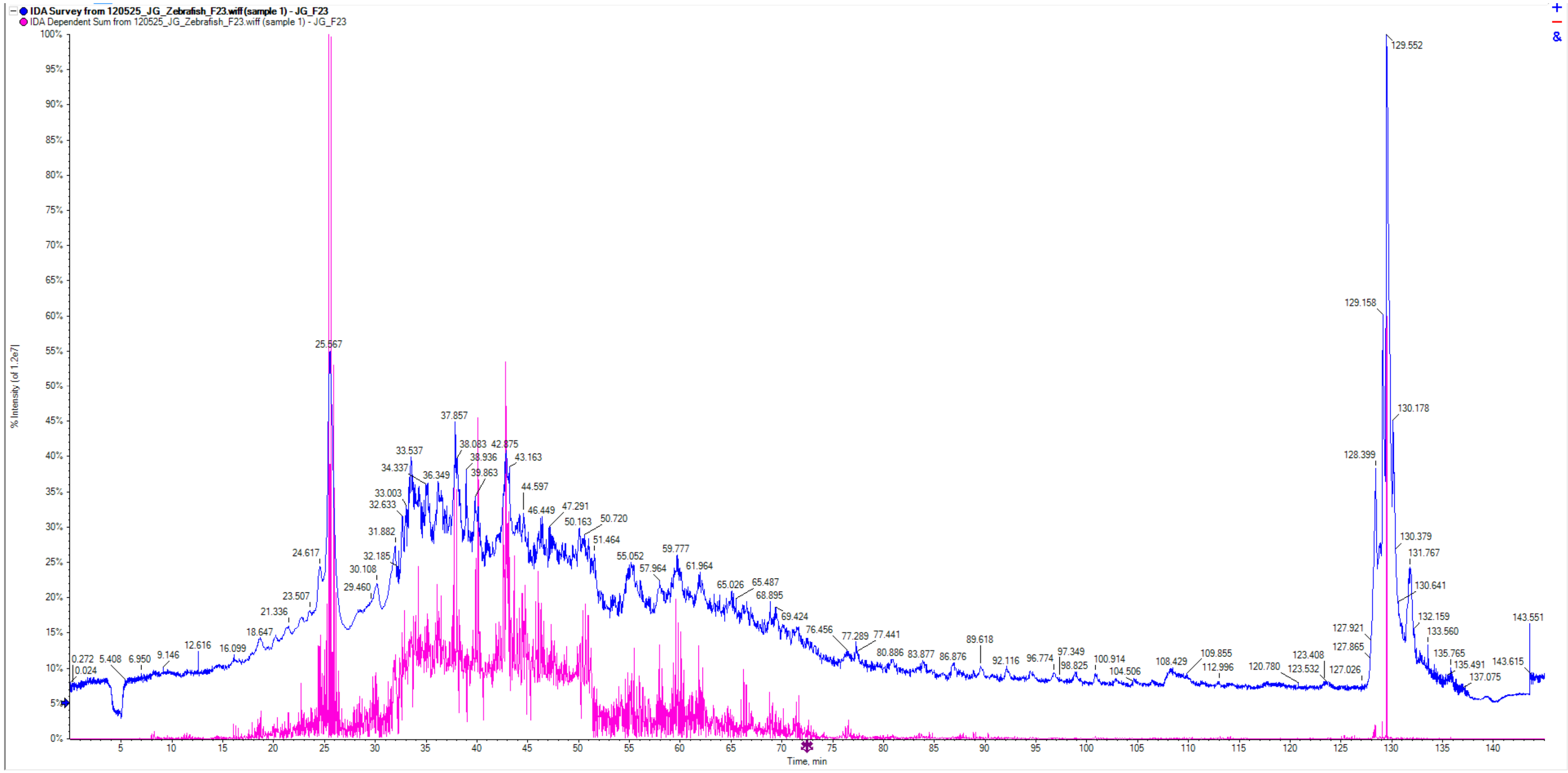

Chromatogram of DOC buffer extracted zebra fish liver sample_Fraction 24 (2D-LC-MS)

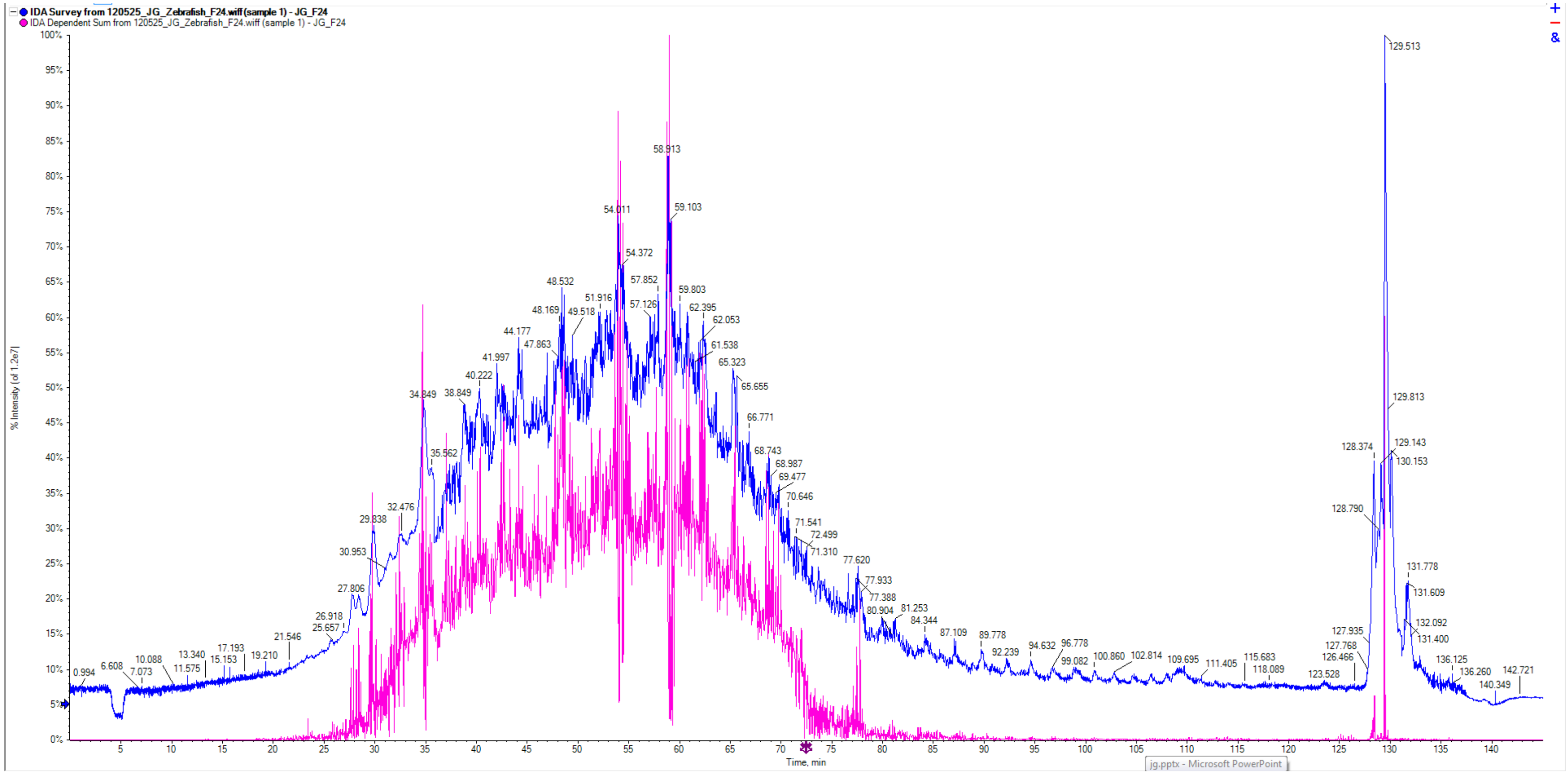

Chromatogram of DOC buffer extracted zebra fish liver sample_Fraction 25 (2D-LC-MS)

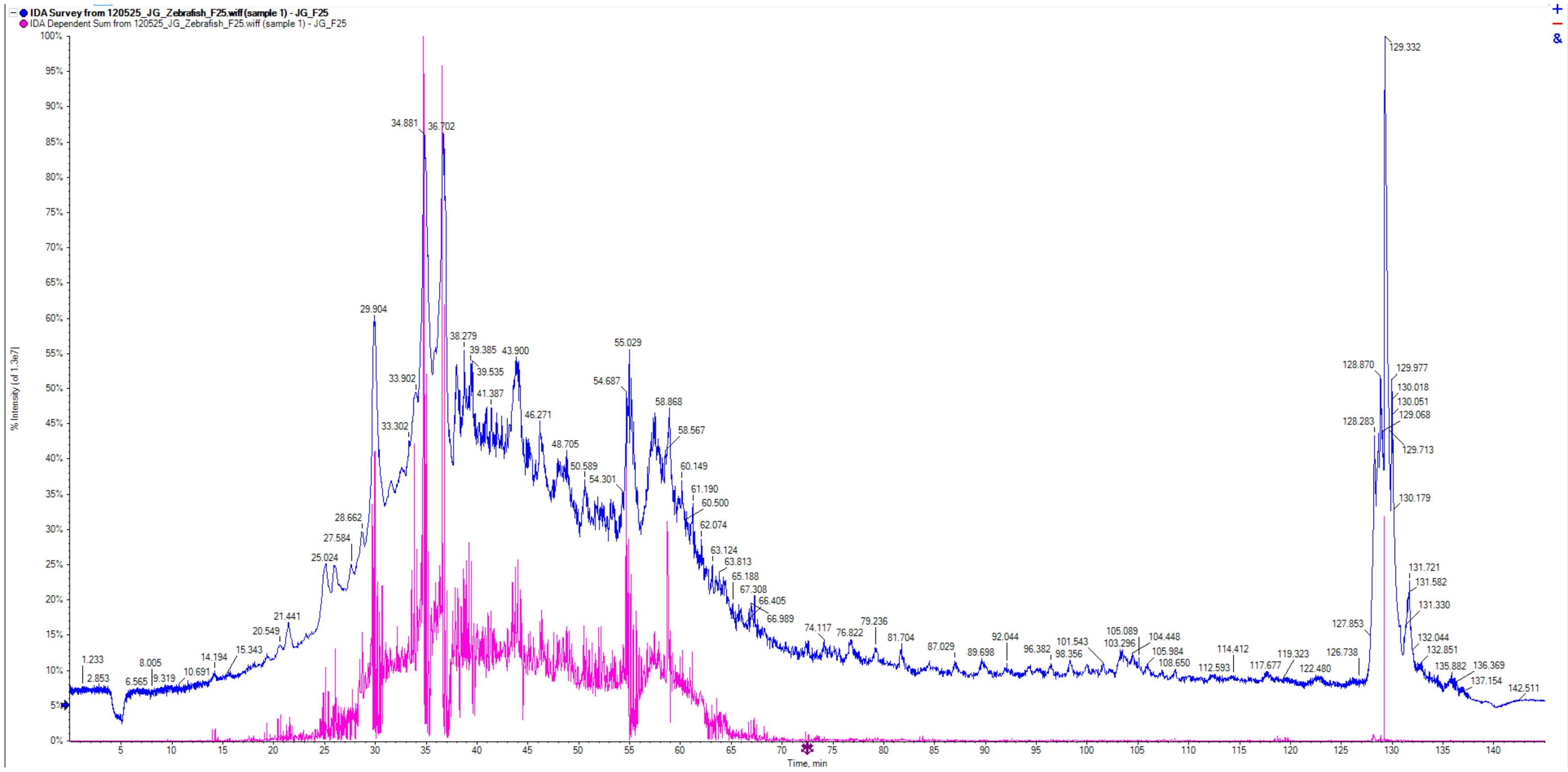

Chromatogram of DOC buffer extracted zebra fish liver sample_Fraction 26 (2D-LC-MS)

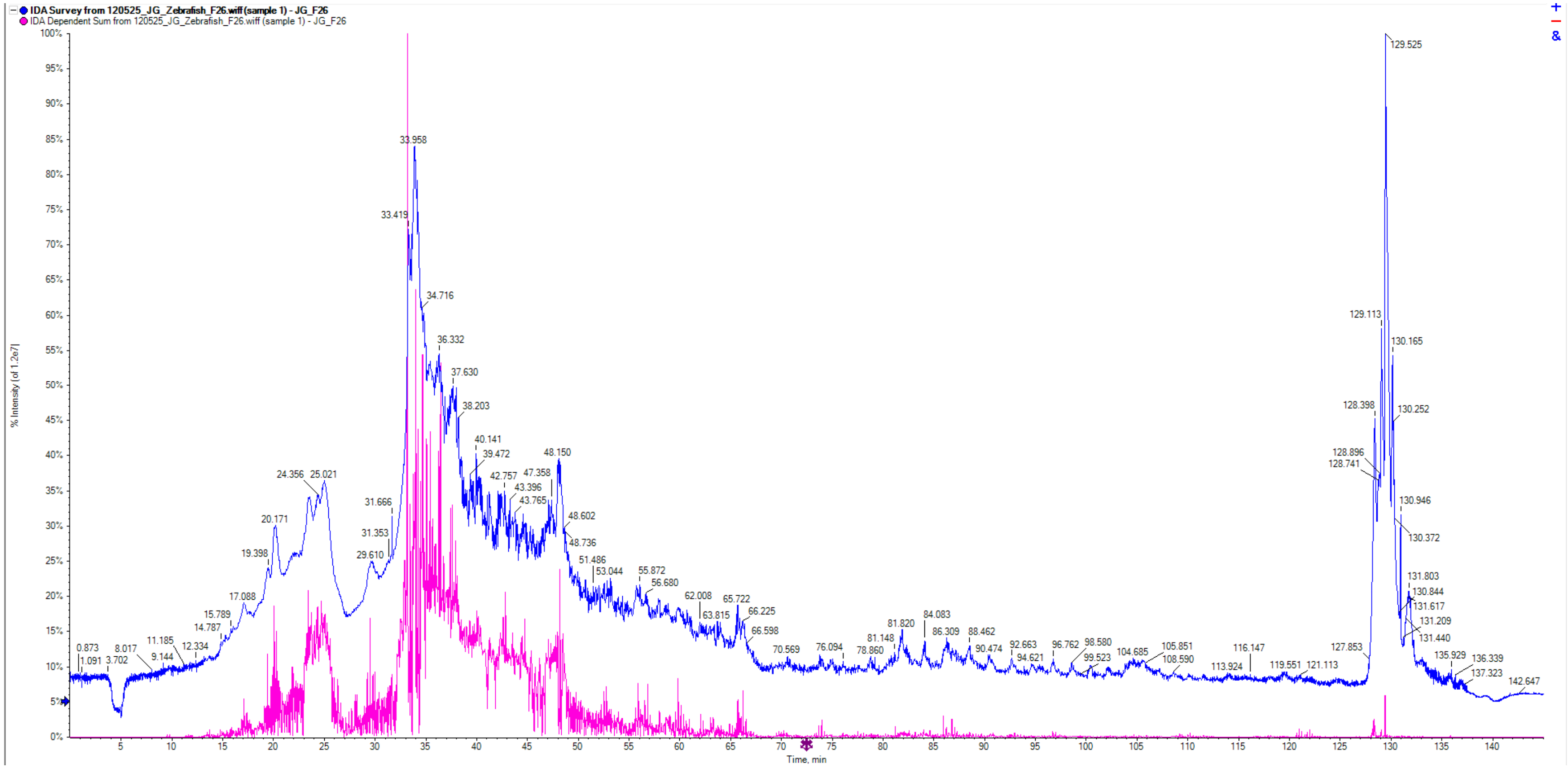

Supplement: Supplementary file 1 — The Supplementary material contains our identified protein list from our 1D shotgun analysis for both DOCΔX (Supplementary Table 1) and SDSΔX (Supplementary Table 2) samples. Supplementary Table 3 contains our identified protein list from our 2D shotgun analysis for DOCΔX sample. Supplementary Table 4 contains the list of representative “canonical pathways” and “diseases and biological functions” with the respective proteins from our identified protein list from Supplementary Table 3 that we obtained from our pathway analysis using IPA. Supplementary Data 1 contains the chromatograms from our 1D (both DOCΔX and SDSΔX) and 2D shotgun analysis (DOCΔX). [file 763969.f1.zip › Supplementary_Data_1.pdf]
